# Progression of white matter hyperintensities is related to blood pressure increases and global cognitive decline – A registered report

**DOI:** 10.1162/imag_a_00188

**Published:** 2024-06-24

**Authors:** Frauke Beyer, Laurenz Lammer, Markus Loeffler, Steffi Riedel-Heller, Stéphanie Debette, Arno Villringer, A. Veronica Witte

**Affiliations:** Bordeaux Population Health Research Center, University of Bordeaux, Inserm, UMR 1219, Bordeaux, France; Department of Neurology, Max Planck Institute for Human Cognitive and Brain Sciences, Leipzig, Germany; Institute for Medical Informatics, Statistics and Epidemiology, University of Leipzig, Leipzig, Germany; Leipzig Research Centre for Civilisation Diseases (LIFE), Leipzig, Germany; Institute of Social Medicine, Occupational Health and Public Health, University of Leipzig, Leipzig, Germany; Day Clinic for Cognitive Neurology, University Hospital Leipzig, University of Leipzig, Leipzig, Germany; CRC 1052 “Obesity Mechanisms”, Subproject A1, University of Leipzig, Leipzig, Germany

**Keywords:** vascular risk, white matter hyperintensities, cognitive aging, neuroimaging, hypertension

## Abstract

White matter hyperintensities (WMH) reflect cerebral small vessel disease (cSVD), a major brain pathology contributing to cognitive decline and dementia. Vascular risk factors, including higher diastolic blood pressure (DBP), have been associated with the progression of WMH yet longitudinal studies have not comprehensively assessed these effects for abdominal obesity or reported sex/gender-specific effects. In this pre-registered analysis of a longitudinal population-based neuroimaging cohort, we investigated the association of baseline DBP and waist-to-hip ratio with WMH progression in linear mixed models. We also examined the relationship of WMH progression and executive and global cognitive function. We conducted gender interaction and stratified analyses. We included data from 596 individuals (44.1 % females, mean age = 63.2 years) with two MRI scans over approximately 6 years. We did not find a significant association of baseline DBP with WMH progression. WMH progression significantly predicted global cognitive decline but not decline in executive function. In exploratory analyses, increases in DBP as well as baseline and increase in systolic blood pressure were associated with WMH progression, confined to frontal periventricular regions. There was no association of WHR nor any gender-specific associations with WMH progression. Adequate BP control might contribute to limit WMH progression and negative effects on global cognitive function in the middle-aged to older population for men and women.

## 1 Introduction

Staying cognitively healthy is of paramount importance when we age and dementia is among the most feared diseases in our society (Hajek & König, 2020). Cerebral small vessel disease (cSVD) has been increasingly recognized as a major underlying pathology of cognitive decline and dementia (Bos et al., 2018). CSVD describes pathologies of the brain’s small arterioles, capillaries, and venules which manifest on magnetic resonance imaging (MRI) as focal lesions (white matter hyperintensities^1^, lacunes, microbleeds, dilated perivascular spaces) and in globally reduced white matter coherence and gray matter atrophy (Wardlaw et al., 2019). Most commonly, WMH volume and location are used as a proxy for cSVD due to relatively easy automatic quantification on brain images. Several studies have shown that the presence and extent of cSVD neuroimaging markers are predictive for stroke, future cognitive decline, and dementia (Debette et al., 2019). While WMH are present in a large proportion of older adults, their occurrence is not random, but their location and extent strongly depends on the presence of vascular risk factors (Jorgensen et al., 2018). It is well known that elevated blood pressure and hypertension are associated with the appearance and progression of WMH in mid and late life (Dufouil et al., 2001;Jansen et al., 2022;Scharf et al., 2019;Vermeer et al., 2007;Williamson et al., 2018; but seeDickie et al., 2016;Sachdev et al., 2007). While both systolic and diastolic blood pressure (DBP) are important predictors, effects seem to be more pronounced for DBP (Zhang et al., 2020). Randomized controlled trials have provided evidence that intensive blood pressure control can reduce the progression of WMH in hypertensive and diabetic patients (de Havenon et al., 2019;Nasrallah et al., 2019;Zhang et al., 2019), yet no consensus on how to specifically target cSVD and related cognitive decline has been reached (Wardlaw et al., 2021).

More recently, abdominal obesity has emerged as a risk factor for cSVD in cross-sectional studies (Higuchi et al., 2017;Kim et al., 2017;Lampe, Zhang, et al., 2019;Morys et al., 2021;Veldsman et al., 2020;Vuorinen et al., 2011;Yamashiro et al., 2014). Mendelian randomization suggested that larger abdominal fat depots (measured as waist-to-hip ratio) are more predictive for WMH than overall obesity (measured as body mass index) (Marini et al., 2020). This effect was largely independent of DBP and glucose metabolism. Along these lines, several studies reported an association between abdominal obesity and WMH in deep white matter regions as opposed to hypertension-related periventricular WMH, hinting to the involvement of different pathophysiological mechanisms (Armstrong et al., 2020;Griffanti et al., 2018;Lampe, Zhang, et al., 2019;Veldsman et al., 2020). One of those mechanisms might be the circulation of systemic inflammatory markers, secreted by abdominal fat tissue, which initiate pathological processes such as endothelial damage and blood brain barrier leakage in the cerebral vasculature of the deep white matter (Wardlaw et al., 2019). Yet, longitudinal evidence is scarce and the RUN-DMC study showed that while high baseline waist circumference predicted a stronger increase in WMH from baseline to follow-up, no predictive effects of continuous waist circumference or body mass index on cross-sectional or longitudinal WMH were found (Arnoldussen et al., 2019). Thus, the impact of abdominal obesity on WMH progression remains to be established.

Self-identified gender, which is assessed in most studies using self-reported binary categories and often misinterpreted as (biological) sex, is another important predictor of WMH. In population-based studies, women tend to show larger and more severe WMH (De Leeuw et al., 2001;Fatemi et al., 2018;Sachdev et al., 2009) while in hospital-based studies, men are overrepresented and show severe cSVD (with stroke or cognitive presentation) more often (Jiménez-Sánchez et al., 2021). Women and men differ in their vascular risk factor profile, for example, the incidence of smoking and hypertension tends to be higher in men, while women tend to develop a more unfavorable abdominal fat distribution after menopause. Additionally, the neuroprotective effects of estrogens are reduced after menopause which might contribute to increased susceptibility of women to neurovascular degeneration and dementia (Dufouil et al., 2014). We therefore hypothesize that higher blood pressure and abdominal obesity might be more strongly associated with WMH progression in women compared to men. Yet, while WMH have been associated with a decline in executive function and other cognitive domains in older adults, their importance for gender-specific cognitive performance is unclear (Kynast et al., 2018). Women have previously not performed worse in cognitive tests despite having higher WMH load (Sachdev et al., 2009).

Therefore, WMH progression might be less negatively associated with cognitive performance in women compared to men. Few studies to date have reported sex/gender-stratified data regarding the association of vascular risk factors and WMH, as well as WMH and cognitive outcomes. This “gender data gap” hampers a better understanding of gender-specific risks and potential prevention strategies. Here, we therefore aim to replicate previous findings on the relationship of higher blood pressure, more WMH progression, and worsening of cognitive function in a large cohort of population-dwelling older adults. In exploratory analyses, we aim to extend these findings towards abdominal obesity, a risk factor which has been understudied in longitudinal designs. We will explore gender-by-risk factor interactions for WMH progression and gender-by-WMH progression interaction for cognitive outcomes. We will also report gender-stratified results for both risk factors if no interaction appears. Finally, we will explore the spatial distribution of new WMH related to the different risk factors.

## 2 Aims and Hypotheses

### 2.1 Confirmatory analyses

Based on the literature and power analyses, we performed replication analyses for the following hypotheses:

- H1: Higher DBP at baseline predicts a stronger increase of WMH volume at follow-up.
- H2: Stronger WMH progression is associated with a stronger decline in executive cognitive function.
- H3: Stronger WMH progression is associated with a stronger decline in global cognitive function.

### 2.2 Exploratory analyses

We tested the following hypotheses in exploratory analyses. These may be underpowered.

- E1a: Higher WHR at baseline predicts a stronger increase of WMH volume at follow-up.
- E1b: Higher change in WHR predicts a stronger increase of WMH volume at follow-up.
- E1c: Higher change in DBP predicts a stronger increase of WMH volume at follow-up.
- E2a: WMH progression is more pronounced in women.
- E2b: There is an interactive effect of gender and DBP on WMH progression, where in women DBP has a stronger effect than in men.
- E2c: There is an interactive effect of gender and WHR on WMH progression, where in women WHR has a stronger effect than in men.
- E3a: There is an interactive effect of gender and WMH progression on executive cognitive function, where in women WMH progression is associated with less decline in executive cognitive function.
- E3b: There is an interactive effect of gender and WMH progression on global cognitive function, where in women WMH progression is associated with less decline in global cognitive function.

If the interactions were not significant, we reported gender-stratified results according to the SAGER guidelines (Heidari et al., 2016).

Finally, we explored the spatial distribution of incident WMH depending on the risk factor profile. For a summary table of planned analysis, seeSupplementary Table 8.

## 3 Methods

### 3.1 Existing data

This project was an analysis in the LIFE-Adult study sample, a longitudinal, two-wave, population-based study conducted in the city of Leipzig, Germany from 2011 until 2021. Baseline characteristics of the LIFE-Adult sample (Loeffler et al., 2015), the baseline association of hypertension and WHR with voxel-wise WMH volume (Lampe, Zhang, et al., 2019), and the cross-sectional link between WMH volume and different cognitive domains (Lampe, Kharabian-Masouleh, et al., 2019) in this sample have been previously published. At the time of the stage-1 protocol, we had access to the baseline anthropometric and medical data and have preprocessed and quality-controlled the imaging data of both time points (bias control level 2). We have not gained access to the follow-up anthropometric, medical, and cognitive data and have not explored any associations of these measures with WMH volume beyond the baseline investigations cited above.

### 3.2 Data availability plan

Due to potential identifiability of individuals from demographic and medical information, we shared a surrogate version of the dataset onhttps://github.com/fBeyer89/VRF-and-progression-of-WMHalong with the analysis code (Nowok et al., 2016). Raw data of the LIFE-Adult cohort can be requested via the LIFE data center (https://ldp.life.uni-leipzig.de/).

### 3.3 Ethics statement

The LIFE-Adult study has been approved by the ethics committee of the University of Leipzig and was conducted according to the declaration of Helsinki. All participants gave written informed consent.

### 3.4 Data collection and preparation

This project is part of a larger population-based epidemiological study LIFE-Adult. LIFE-Adult has investigated 10,000 individuals from the Leipzig area, who underwent genotyping and deep phenotyping at up to two time points (including extensive questionnaires, MRI, and cognitive testing in a subgroup of N ~2700). Recruitment and inclusion criteria as well as more information on the study design and objectives can be found inLoeffler et al. (2015)andEngel et al. (2023). Baseline assessments took place from 2011 to 2014, and the follow-up visits were scheduled between 2017 and 2021. For the follow-up visit, participants from the LIFE-Adult MRI cohort (Nbaseline = ~2700) were re-invited to participate in medical assessments, cognitive testing, and MRI scanning. In total, 1077 participants underwent MRI at follow-up. For this analysis, we included all participants who were aged between 45 and 85 years at the baseline assessment based on recent studies showing WMH volume to increase from the fifth life decade on (d’Arbeloff et al., 2019;Wen et al., 2009). All included participants were scanned twice with a mean time between scans of 6 years (standard deviation = 1.9 years).

### 3.5 Anthropometrics

Waist and hip circumferences were taken by trained study staff using an ergonomic circumference measuring tape (SECA 201) to the nearest 0.1 cm at baseline and follow-up. WHR was calculated by dividing waist by hip circumference. We used baseline WHR and change in WHR, calculated as difference between follow-up and baseline (i.e., WHR_change = WHR_followup – WHR_baseline), as independent variables of interest.

### 3.6 Blood pressure

Diastolic blood pressure was measured three times at 3-minute intervals using an automatic oscillometric blood pressure monitor (OMRON 705IT, OMRON Medizintechnik Handelsgesellschaft mbH) in participants seated for at least 5 minutes at baseline and follow-up. We will calculate the average of the three DBP measurements for our analysis. We used baseline DBP and change in DBP, calculated as difference between follow-up and baseline (i.e., DBP_change = DBP_followup – DBP_baseline), as independent variables of interest.

### 3.7 Cognitive assessment

In both LIFE-Adult assessments, participants underwent the Consortium to Establish a Registry for Alzheimer’s Disease (CERAD) -plus test-battery, an established set of neurocognitive tests designed to detect early cognitive changes related to Alzheimer’s disease (AD) (Morris et al., 1989). It includes a word list testing verbal memory and a test of semantic fluency (Animals). The applied version additionally includes the Trail-Making-Test (TMT) and phonemic fluency (S-words) to assess executive function and verbal fluency independent of semantic memory. We derived a composite score of executive function and a global cognitive score similar to previous studies (Beyer et al., 2017;Kharabian Masouleh et al., 2016;Oosterman et al., 2010). The executive function summary score was calculated as the sum of z-scored time to complete TMT part B over time to complete TMT part A, phonemic and semantic fluency (verbal fluency). Z_exec = [– z (time for TMT part B/time for TMT part A)+ z_phonemic fluency + z_semantic fluency]/3 The global score was based on the executive function score, processing speed, and a composite memory score. The processing speed score was given by the Z-scored negative value of the time taken to complete part A. Z_processing_speed = -z(TMT time for part A). For the memory score, we used learning, recall, and recognition from the CERAD word list. Learning was defined as the sum of three consecutive learning trials of the CERAD word list (10 words), recall as the sum of correctly recalled words after a delay, in which participants performed a nonverbal task, and recognition as the number of correctly recognized words out of a list of 20 presented afterwards. Z_memory = (z_sum_learning + z_recall + z_recognition)/3. The global cognitive performance score was derived by summing up the z-scores from all four domains: Z_global_cognition = Z_exec + Z_proc + Z_memory/3. All individual sub-scores were Z-scored across time points prior to creating composite scores. The composite scores for executive function and global cognition were again Z-scored.

### 3.8 Imaging acquisition and preprocessing

At baseline and follow-up, anatomical and lesion-sensitive imaging was acquired on a 3T MAGNETOM Verio scanner (Siemens, Erlangen, Germany) with a 32-channel head coil. Anatomical imaging was done with a T1-weighted magnetization prepared rapid acquisition gradient echo (MPRAGE) sequence with the following parameters (flip angle = 9 degrees, relaxation time [TR] = 2300 ms, inversion time [TI] = 900 ms, echo time [TE] = 2.98 ms, 1-mm isotropic resolution, acquisition time [AT] = 5.10 minutes), and the lesion-sensitive imaging was performed with a fluid-attenuated inversion-recovery (FLAIR) sequence (TR = 5000 ms, TI = 1800 ms, TE = 395 ms,1 × 0.49 × 0.49 mm resolution, AT = 7.02 minutes).

#### 3.8.1 Lesion segmentation

The longitudinal pipeline of the Lesion Segmentation Toolbox (version 3.0.0, run on MATLAB version 9.10) was used to estimate WMH progression (Schmidt & Wink, 2017). This pipeline estimates the location of stable lesions as well as regression and progression of lesions over time (Schmidt et al., 2019). First, we performed cross-sectional lesion segmentation using the Lesion Prediction algorithm with its default parameters. Then, we applied the longitudinal pipeline to the cross-sectional runs and obtained voxel-wise maps of lesion change (LCL maps). In these three-valued whole-brain maps, 1 indicates a regression of lesion volume, 2 indicates a stable lesion, and 3 indicates a newly appeared lesion in this voxel. For baseline lesion volume, we summed up the volume of all LCL voxels with a value of 2 and for follow-up lesion volume, we added the volumes of all LCL voxels with a value of 1 (regressed lesion voxels) or 3 (novel lesion voxels). For our analysis, we calculated asinh-transformation of baseline WMH volume (asinh(WMHBL)) and change as difference of asinh-transformed WMH volume at follow-up and baseline (WMHchange=asinh(WMHFU)-asinh(WMHBL)) to achieve a normal distribution of regression residuals. During visual quality control, we checked whether the lesions marked in the LCL were confounded due to poor scan quality, lesion regression, or brain pathologies at baseline or follow-up. We gave the following LCL quality ratings: issues with MRI data quality, for example, due to motion (LCL quality = 1), ventricular expansion which led to regression of lesion voxels in some cases (LCL quality = 2), and brain pathologies such as stroke or congenital lesions (LCL quality = 3).

#### 3.8.2 Anatomical preprocessing

T1-weighted imaging was processed with the longitudinal stream of FreeSurfer version 5.3.0 to derive estimated total intracranial volume (TIV) (Reuter et al., 2012). We z-scored the value to achieve a more stable model fitting.

### 3.9 Medical, demographic, and questionnaire data

Participants were asked to report previous cardiovascular and other diseases as well as the intake of medication. Self-reported medication was classified according to the Anatomical Therapeutic Chemical (ATC) Classification System. The intake of anti-hypertensive medication was defined based on self-reported intake of hypertensive medication in the cardiological questionnaire or the intake of anti-hypertensive medication based on the list of medication (seeSupplementary Table 8). Here, we used ATC codes starting with “C02,”C03”, “C07”, “C08”, “C09” as indicators of anti-hypertensive medication. The use of centrally active medication was defined based on the self-reported intake of medication with the ATC codes M03B (muscle relaxants, centrally acting agents), N02A (opioids), N03 antiepileptics, N04 anti-parkinson drugs, N05 psycholeptics, N06A antidepressants, N06B psychostimulants, agents used for ADHD and nootropics, N06D anti-dementia drugs (except for N06Dx02, ginkgo folium), or N07A parasympathomimetics (seeSupplementary Table 8). Participants underwent the SIDAM (structured interview for the diagnosis of dementia) which includes the Mini Mental State Examination (MMSE) at baseline and follow-up. Self-reported level of education was dichotomized into a binary variable indicating the attainment of tertiary education (Lampert et al., 2013). We used 3.6 as cut-off. Education was only assessed at baseline. For the assessment of depressive symptoms, participants filled in the German version of the Center for Epidemiological Studies-Depression scale at baseline and follow-up. We derived the summary score ranging from 0 to 60.

### 3.10 Data exclusion

We excluded participants with neurological or psychiatric disease at baseline or follow-up (i.e., radiological finding of ischemic, traumatic, or hemorrhagic lesion in MRI, incidental finding leading to non-usability of participant, multiple sclerosis, Parkinson’s disease, epilepsy, previous stroke, self-reported dementia, intake of centrally active medication, or a score of <24 in the MMSE; seeSupplementary Table 8). If participants lacked information on these variables for one or both time points, we did not exclude the participant. Only participants with complete longitudinal WMH data were included. Further, participants for whom the Lesion Segmentation Toolbox did not run correctly or who were labeled to have poor scan quality or brain pathologies (LCL quality = 1 or 3 ) during quality control were excluded from all analyses (H1–H3 and exploratory analyses). Time points with extreme outliers in TMT A (time to complete over 300 s) and B (time to complete over 300 s) were not considered in the analysis of executive function score (H2). Participants who missed WHR or DBP or had biologically implausible values (see below) at baseline and follow-up were excluded from the analysis. Otherwise, biologically implausible values in waist-to-hip ratio (<0.5 or >1.5) or blood pressure (DBP>140 mmHg and SBP<DBP) were imputed (see below).

### 3.11 Missing data

#### 3.11.1 Dependent variables

Only participants with complete and usable WMH data at both time points were investigated. If participants missed data on any of the cognitive tests (e.g., TMT, semantic or phonemic fluency, CERAD word list), we constructed the executive function and global composite score from the remaining tests. If participants did not have data on any test for executive function (i.e., no data on TMT, phonemic or semantic fluency) or global composite score (i.e., no cognitive data at all) for both time points, they were excluded for the respective analyses. Otherwise, default listwise deletion of the respective time point was performed in the mixed models.

#### 3.11.2 Independent variables

If participants were missing WHR or DBP at only one occasion, we imputed the missing value. If participants missed the measures or had biologically implausible values (see above) at both time points, they were excluded from the analysis.

#### 3.11.3 Covariates

If participants were missing information on education (assessed only at baseline), hypertensive treatment, CES-D at one or both time points, or TIV, we imputed the missing values.

#### 3.11.4 Imputation

Multi-level imputation was performed with the R package mice 3.9.0 for education, TIV, DBP, WHR, CESD, and hypertensive treatment (see prepare_data.R on github). The imputation was based on all available cases after applying exclusion criteria and was repeated five times with 10 iterations. We reported the percentage of missing data for each of the variables. Imputation methods for education and TIV (2nd-level variables) were “2l.bin” and “2lonly.pmm”, and for DBP, WHR, CESD, and hypertensive treatment we used “2l.pan”. SeeTable 1for the variables used for the imputations.

**Table 1.** Missing values in rows (education, TIV, DBP, WHR, CESD, and hypertensive treatment) will be imputed based on the variables marked in the columns.

Predicted variable	Subject ID	Time	Age	Gender	Education	TIV	DBP	WHR	CESD	Hypertensive treatment
Education	X		X	X						
TIV	X		X	X						
DBP	X	X	X	X	X			X	X	X
WHR	X	X	X	X	X		X		X	X
CESD	X	X	X	X	X		X	X		X
Hypertensive treatment	X	X	X	X			X	X		

### 3.12 Power calculation

#### 3.12.1 Power calculation for model M1

We performed a power calculation by simulating the effects of interest based on LIFE-Adult baseline data and previous studies. All code can be found onhttps://github.com/fBeyer89/VRF-and-progression-of-WML.

We simulated individual data points based on three components: cross-sectional variation, longitudinal variation, and error terms.

We based the cross-sectional variation on the baseline associations of age, gender, systolic blood pressure (SBP), and WHR with WMH in LIFE-Adult participants over 50 years (Table 2). We used systolic blood pressure but effects have been shown to be similar or more pronounced for DBP. First, we fitted the predictors to the baseline WMH load using a log-linked GLM from the Gamma family.

**Table 2.** Cross-sectional estimates of age, systolic blood pressure (SBP), waist-hip ratio (WHR), gender, intracranial volume (ICV), and WMH volume.

N = 1574	Estimate	Standard error
Intercept	1.44	0.09
Age	0.067	0.007
SBP	0.011	0.003
WHR	2.15	0.74
Gender (male)	-0.40	0.15
ICV	0.000002	0.0000007

Legend: These estimates from the baseline assessment were used for the power analysis only.

The advantage of this approach is that we could use these coefficients to estimate WMH load in its original unit (cm^3^) and thus combined cross-sectional effects with longitudinal effect sizes from the published literature. Then, we drew random samples from a multivariate normal distribution of age, gender, SBP, WHR, and ICV with the same mean and covariance matrix as in the baseline data. Using the coefficients derived from the GLM and the simulated predictors, we calculated baseline estimates of WMH in cm³.

The longitudinal effect of elapsed time on WMH was based on eight epidemiological and interventional studies in older adults (age > 60 years) (de Havenon et al., 2019;Dickie et al., 2016;Godin et al., 2011;Nasrallah et al., 2019;Peng et al., 2014;Scharf et al., 2019;Schmidt et al., 2005;Table 3). The weighted average annual change in WMH based on these studies was 0.64 cm³. As the prevalence of risk factors (hypertension, diabetes) and mean age varies across these studies, an average WMH annual change of 0.64 cm³ is likely to overestimate the isolated effect of time on WMH. Further, most studies reported the estimates in units of cm³ from linear models without considering the strongly skewed distribution of WMH volume, and are thus biased. For a more conservative estimate, we based the individual change in WMH from baseline to follow-up on a normal distribution with the mean at the half of the estimated WMH annual change (0.32 cm³/y) and a relatively low standard deviation of 0.1 cm³, reflecting the fact that elapsed time is overall positively associated with the progression of WMH. If values of age-related WMH change below zero were drawn, they were set to 0.01.

**Table 3.** Publications used for the effect of time on WMH volume.

Publication	Type of study, number of participants	Time between time points	Effect size of time on WMH volume Mean (SD) of annual increase or point estimates
Scharf et al. (2019)	Epidemiological study N = 554	3 years	60-69y: 0.54 (1.27) cm³/y n = 24770-79y: 1.04 (1.93) cm³/y n = 18680 +: 1.6 (2.4) cm³/y n = 121
Dickie et al. (2016)	Cohort study N = 439	3 years	11.9 ± 11.7 cm³ at 73 years 15.9 ±14.6 cm³ at 76 years
Godin et al. (2011)	Epidemiological study N = 1319	4 years	1.07 (2.76) cm³ over 4 years
Peng et al. (2014)	Epidemiological study of hypertensive patients N = 294	4 years	Baseline: 13.78 cm³+-6.67 Follow-up: 17.82 cm³ +-8.74
Schmidt et al. (2005)	Epidemiological study N = 243	6 years	1.38 (3.76 ml) cm³
Maillard et al. (2009)	Epidemiological study N = 1118	4 years	0.25 (0.56) cm³/y
Nasrallah et al. (2019)	Intervention study, hypertensive patients from standard treatment group N = 200	3.98 years	1.45 cm³
de Havenon et al. (2019)	Intervention study, diabetic patients in the glycemic intervention arm N = 502	40 months	0.93 ± 1.20 cm³

The modifying effect of baseline SBP and change in SBP on age-related change in WMH load was based on four epidemiological studies (Dickie et al., 2016;Godin et al., 2011;Gottesman Rebecca et al., 2010;Verhaaren et al., 2015;Table 4). For baseline SBP, the average modifying effect of 1 mmHg average SBP was 0.0052 cm³/y. We used a standard deviation of 0.001 cm³/y to draw change estimates due to baseline SBP from a normal distribution. The effect of change in SBP could be drawn from only one study (Godin et al., 2011) and was 0.0025 cm³/y per mmHg. Again, we used a normal distribution with a standard deviation of 0.001 cm³/y.

**Table 4.** Studies used to estimate the longitudinal effects of baseline SBP and change in SBP on WMH progression.

Publication	Type of study, number of participants	Time between time points	Effect size of baseline SBP on WMH progression
Godin et al. (2011)	Epidemiological studyN = 1319	4 years	0.04 (0.02) cm³ per 5 mmHg
Dickie et al. (2016)	Cohort studyN = 439	3 years	0.0271 cm³
Gottesman Rebecca et al. (2010)	Epidemiological studyN = 983	6 years	1.1 cm³ in 10 years /20 mmHg
Verhaaren et al. (2013)	Epidemiological studyN = 1118	~4 years	0.08 (0.03; 0.14) cm³/y per SD of SBP SD = 18 mmHg

Publication	Type of study, number of participants	Time between time points	Effect size of change in SBP on WMH load
Godin et al. (2011)	Epidemiological studyN = 1319	4 years	0.05 (0.02) cm³ per 5 mmHg SBP increase

Previous longitudinal studies did not investigate baseline WHR as a predictor of WMH progression. Studies on BMI either reported no effect (Dearborn et al., 2015;Scharf et al., 2019) or did not show quantitative effect sizes (Gustafson et al., 2004;Vuorinen et al., 2011). Yet, cross-sectional studies indicate that WHR is associated with WMH, predominantly in deep WM (Alqarni et al., 2021;Griffanti et al., 2018;Higuchi et al., 2017;Kim et al., 2017;Lampe, Zhang, et al., 2019;Morys et al., 2021;Veldsman et al., 2020). Thus, while there are little longitudinal data to rely on, based on cross-sectional reports we expect a smaller effect size for WHR compared to blood pressure.

We obtain an exploratory estimate of the effect size by comparing the baseline association in the LIFE-Adult cohort of SBP and WHR with asinh-transformed WMH volume. Here, the coefficients are 0.84 (asinh(cm³))/WHR unit and 0.0083 (asinh(cm³)/mmHg) for WHR and SBP, respectively. We use the approximation that the interaction effect of WHR on age change would be similar to the interaction effect of SBP (0.0052 cm³/y), scaled by their ratio, leading to an interaction effect of WHR of 0.0052 cm³/y/mmHg * 0.84/0.0083 = 0.53 cm³/y. This approach is not ideal as it combines effect sizes from the literature referring to raw WMH units (cm³) with relationship of effects on log-scaled data. Yet, it is the best we can do given the lack of appropriate data on the expected effect size.

In our simulations, we will thus estimate the power for a range of scales of this exploratory effect size (0.5, 1, and 1.5 times 0.53 cm³/y). We will use the same values for the effect of change in WHR. Changes in SBP and WHR from baseline to follow-up were based on published results in epidemiological studies of aging (Baltimore Longitudinal Study of Aging (BLSA) and Whitehall II). Average time between both assessments in LIFE-Adult was 6.7 years.

We estimated the average change in SBP to be: 0.76 mmHg/y (averaged over BLSA: 8.5 mmHg/decade for men, 4.4. mmHg/decade for women at age 60 and Whitehall 2: 1 mmHg/y for older men/women (60–70 years)) (Dearborn et al., 2015;Wills et al., 2011). We thus drew the change in SBP from a normal distribution with a mean of 0.76 mmHg/y * 6.76y = 5.13 mmHg and arbitrary, yet relatively high standard deviation of 4 mmHg. For WHR,Shimokata et al. (1989)reported an increase of WHR of 0.0073 in men, 0.0021 in women over 5 years. Thus, WHR change was taken from a normal distribution with a mean of 0.0047/5 6 0.0056 and a similarly high standard deviation of 0.005.

For the error terms, we used a subject random effect with a mean of zero and a standard deviation of 0.5 cm³, while for the random error we used a normal distribution around zero with 1 cm³ standard deviation.

Finally, all effects were added according to

*WMH=*

exp(age_sim*coeff_age +….)(cross-sectional effects from Gamma-loglink GLM)

+(effect_age_change+((effect_SBP_baseline*SBP_baseline)+(effect_WHR_baseline*WHR_baseline)*age_change)(effects of elapsed time/change in age, modified by baseline SBP and WHR)

*+ WHR_*change*effect_WHR_change + SBP_change*effect_SBP_change(effects of change in SBP and WHR)

+ random_effect + residual_error (**residual error and random effects)**

Then, we repeated the simulation 50 times for four sample sizes (N = 400, 600, 800,1000) and for three scaling factors of WHR effects (0.5, 1, 1.5).

We used the asinh-transform and fitted the linear mixed model M1. We extracted p-values for the interaction effects of SBP baseline, and WHR baseline on the age change effect, as well as the effects of SBP and WHR change, and considered p < 0.033 as significant. Then, we derived the power by calculating the number of rejected null hypotheses compared to the total number of tests. If the average effect size across simulations was not in the expected direction (positive for all four predictors), we assigned a power of 0.

We also extracted the average Bayes Factor and one-sided Bayes Factor (based on 10 Markov chains to calculate proportion of posterior estimates in the hypothesized directionTable 5andTable 6).

**Table 5.** Simulated power (α < 0.05, one-sided tests) to detect an interaction effect of baseline SBP and WHR with age change and effects of change in SBP and WHR on progression of WMH.

Sample size	Interaction of SBP baseline with age change	Interaction of WHR baseline with age change	SBP change	WHR change	WHR factor
400	0.62	0	0	0	0.5
600	0.9	0	0.02	0	0.5
800	0.98	0	0	0	0.5
1000	1	0	0.02	0	0.5
400	0.78	0	0.04	0.02	1
600	0.96	0	0.02	0.02	1
800	0.98	0	0.02	0	1
1000	0.98	0	0.04	0.04	1
400	0.84	0	0.02	0	1.5
600	0.86	0.1	0.04	0.02	1.5
800	1	0.16	0.06	0	1.5
1000	1	0.12	0.04	0	1.5

**Table 6.** Simulated average one-sided Bayes Factors for the interaction effect of baseline DBP and baseline WHR with age change and SBP and WHR change on progression of WMH.

Sample size	Interaction of DBP baseline with age change	Interaction of WHR baseline with age change	DBP change	WHR change	WHR factor
400	1.15	0	0	0	0.5
600	3.68	0	0	0	0.5
800	18	0	0.05	0	0.5
1000	40.3	0	0.05	0	0.5
400	1.10	0	0	0	1
600	4.26	0	0	0	1
800	8.80	0	0.06	0	1
1000	8.33	0	0	0	1
400	2.26	0.09	0	0	1.5
600	1.94	0	0	0	1.5
800	39.4	0.09	0	0	1.5
1000	78.4	0	0	0	1.5

We thus concluded that after applying exclusion criteria to our sample of N ~ 1000 individuals, we would be able to detect the interaction of DBP with age change with a power > 0.9 and a Bayes Factor > 10. We would not be sufficiently powered to detect the hypothesized effect size of baseline WHR on WMH progression. We thus report these results in the exploratory analysis section. Similarly, we would not be sufficiently powered to detect effects of change in DBP and WHR on WMH progression and also reported these results in the exploratory analysis section Power calculation for Model M2 and M3.

A negative effect of WMH progression on executive and global cognitive function is well established in non-clinical populations (Debette et al., 2019;Hamilton, Backhouse, et al., 2021;Kloppenborg et al., 2014).

Unfortunately, effect sizes for WMH progression have rarely been reported in quantitative units but have been calculated for semi-quantitative ratings or dichotomized quantitative outcomes. Thus, we base the following power analysis on a recent investigation in 540 members of the Lothian Birth cohort (average age: 72.6 years) over 9 years (Hamilton, Backhouse, et al., 2021).

Here, the ratio of WMH load normalized by TIV predicted a decline in global cognitive function (standardized β = −0.149) and processing speed (standardized β = −0.176).

As our cohort is younger on average than the Lothian Birth cohort, we expected the effect size to be smaller, yet still reliable. Using the pwr package in R, we estimated a minimum number of 850 participants to detect a small negative effect of WMH volume on cognitive function (standardized β = −0.1; pwr.r.test (r = −0.1, sig.level = 0.05, power = 0.9, alternative=“less”)) and a minimum number of 590 participants for a slightly larger effect (standardized β = −0.12; pwr.r.test (r = −0.1, sig.level = 0.05, power = 0.9, alternative=“less”)).

Our power should thus be sufficient to detect the effect on global and executive cognitive function in our cohort (Table 7).

**Table 7.** Summary table with an overview of research questions, hypotheses, planned analyses, and interpretation of outcomes.

Question	Hypothesis	Sampling plan	Analysis plan	Rationale for deciding the sensitivity of the test for confirming or disconfirming the hypothesis	Interpretation given different outcomes	Theory that could be shown wrong by the outcomes
Does diastolic blood pressure predict WMH progression?	H1: Higher diastolic blood pressure at baseline is associated with a stronger increase in WMH progression.	See section “ Power Calculation ”	Statistical model:M1:asinh(WMH) ~ Age_baseline + Age_change + DBP_baseline + **DBP_baseline:Age_change** + DBP_change + WHR_baseline + WHR_baseline:Age_change + WHR_change + gender + HT_medication + TIV + (1|subj)Inference:Frequentist/Bayes Factor analysis comparing M1 with a null model leaving out the term “ **DBP_baseline:Age_change”**	p < 0.033 and BF > 6 If p < 0.033 and BF > 3 p < 0.033 and BF > 1/3 and BF < 3 p > 0.033 and BF > 1/3 <3 p > 0.033 and BF < 1/3 p > 0.033 and BF < 1/6	positive evidence for H1 moderate evidence for H1 weak evidence for H1 inconclusive evidence **moderate evidence for H0** positive evidence for H0	Diastolic blood pressure is a risk factor for progression of WMH.
Is WMH progression associated with a decline in executive function?	H2: A stronger increase in WMH volume from baseline to follow-up is associated with a stronger decrease in executive function.	See section “ Power Calculation ”	Statistical model:M2:Z_exec ~ asinh(WMH)_baseline + **WMH_change** + Age_baseline + Age_change:asinh(WMH)_baseline + Age_change + gender + education + CESD + (1|subj)Inference:Frequentist/Bayes Factor analysis comparing M2 with a null model leaving out the term “ **WMH_change”**	p < 0.033 and BF > 6 If p < 0.033 and BF > 3 p < 0.033 and BF > 1/3 and BF < 3 p > 0.033 and BF > 1/3 < 3 p > 0.033 and BF < 1/3 p > 0.033 and BF < 1/6	positive evidence for H1 moderate evidence for H1 weak evidence for H1 **inconclusive evidence** moderate evidence for H0 positive evidence for H0	MRI markers of cSVD are associated with specific cognitive decline.
Is WMH progression associated with a decline in general cognitive function?	H3: A stronger increase in WMH volume from baseline to follow-up is associated with a stronger decrease in global cognition.		M3:Z_globalcog ~ asinh(WMH)_baseline + **WMH_change** + Age_baseline + Age_change:asinh(WMH)_baseline+ Age_change + gender + education + CESD + (1|subj)Inference:Frequentist/Bayes Factor analysis comparing M3 with a null model leaving out the term “ **WMH_change”**	p< 0.033 and BF > 6 If p < 0.033 and BF > 3 p < 0.033 and BF > 1/3 and BF < 3 p > 0.033 and BF > 1/3 < 3 p > 0.033 and BF < 1/3 p > 0.033 and BF < 1/6	**positive evidence for H1** moderate evidence for H1 If weak evidence for H1 inconclusive evidencemoderate evidence for H0 positive evidence for H0	MRI markers of cSVD are associated with general cognitive decline

Legend: In column “Interpretation given different outcome”, interpretations supported by the data are highlighted in bold.

### 3.13 Statistical analysis

#### 3.13.1 Confirmatory analyses

Accepted stage 1 registered report protocol can be found inhttps://osf.io/7jafe. Statistical analyses scripts can be inspected onhttps://github.com/fBeyer89/VRF-and-progression-of-WML. All statistical analysis with WMH volume or executive function as dependent variable was performed in R version 4.2.2. We used linear mixed models implemented in lmerMod (lmerTest) and BayesFactor version 0.9.12-4.2 (generalTestBF) with subject as a random intercept (see function run_LME_realdata.R on github).

More specifically, we tested three models for our four hypotheses (seeTable 8). M1: asinh(WMH) ~ Age_baseline + Age_change + DBP_baseline + DBP_baseline:Age_change + DBP_change + WHR_baseline + WHR_baseline:Age_change + WHR_change + gender + HT_medication + TIV + (1|subj)

**Table 8.** Baseline and follow-up demographic characteristics of participants included in the study.

	Baseline			Followup		
	N	Mean	SD	N	Mean	SD
Age (y)	596	63.2	8.94	596	69.9	8.87
Gender (female)	596	263 (44.1%)		596	263 (44.1%)	
**Education (tertiary education)**	593	313 (52.8%)		593	313 (52.8%)	
**DBP (mmHg)**	590	76.3	9.33	592	76.5	9.52
**WHR**	595	0.941	0.0855	593	0.943	0.0929
**CESD**	554	2.64	0.8	558	2.55	0.92
**Antihypertensive Medication (yes)**	595	261 (43.9%)		538	341 (63.4%)	
WMH volume (cm³)	596	1.88	3.87	596	3.03	5.22

Legend: Variables in bold had missing values and were imputed. Complete sample size was N = 596. DBP: diastolic blood pressure, WHR: waist-to-hip ratio, CESD: center for epidemiological studies depression scale,WMH: white matter hyperintensities, SD: standard deviation.

M2: Z_Exec ~ Age_baseline + Age_change + asinh(WMH)_baseline + Age_change:asinh(WMH)_baseline + WMH_change + gender + education + CES_D

M3: Z_global_cog ~ Age_baseline + Age_change + asinh(WMH)_baseline + Age_change:asinh(WMH)_baseline + WMH_change + gender + education + CES_D

**Explanation of covariates (M1)**

- Age_baseline: effect of age at baseline
- Age_change: effect of passed time between baseline and follow-up (progression)
- DBP_baseline: effect of baseline DBP
- DBP_baseline: modifying effect of baseline DBP on progression of WMH between baseline and follow-up (effect of interest for H1)
- DBP_change: effect of change in DBP between baseline and follow-up on WMH progression (effect of interest for E1c)
- WHR_baseline effect of baseline WHR
- WHR_baseline:Age_change: modifying effect of baseline WHR on progression of WMH between baseline and follow-up (effect of interest for E1a)
- WHR_change: effect of change in WHR between baseline and follow-up on WMH progression (effect of interest for E1b)
- Gender: adjust for gender (no power analyses possible for gender/sex interaction, therefore we control for it in confirmatory analyses)
- HT_medication: adjust for hypertension medication as this probably influences the effect of DBP on WMH progression
- TIV: total intracranial volume, trivially linked with WMH volume

**Explanation of covariates (M2 & M3)**

- Age_baseline: effect of age at baseline
- Age_change: effect of passed time between baseline and follow-up (progression)
- asinh(WMH)_baseline: effect of baseline WMH volume
- Age_change:asinh(WMH)_baseline: modifying effect of baseline WMH load on cognitive function changes between baseline and follow-up
- WMH_change: effect of interest M2/M3: effect of WMH progression on cognitive function changes
- Gender: adjust for gender (no power analyses possible for gender/sex interaction, therefore we control for it in confirmatory analyses)
- Education: adjust for education level as it probably influences overall cognitive performance
- CES-D: adjust for depressive symptoms as they influence overall cognitive performance

#### 3.13.2 Inference criteria

We based our inference on frequentist and Bayesian full null model comparison. For frequentist statistics, we used the two-sided p-value for the fixed effects calculated in lmerTest using Satterthwaite’s denominator degrees of freedom. Additionally, we applied a multivariate Wald test implemented as D1 in mice to compare models with and without the terms of interest age x DBP baseline and WMH change for multiple imputations. As we have directed hypotheses, we used one-sided p-values (αTwoSided = 2 * αOneSided). We will Bonferroni-adjust for three tested hypotheses by dividing the alpha-level of 0.05 by 3 (αOneSided < 0.05/3). Practically, we used αTwoSided = 2* αOneSided /3 = 0.033 as threshold on the two-sided p-values we received from lmerTest and D1. To obtain Bayes Factors, we fit generalTestBF with the options whichModels=“top”, multicore = T, neverExclude = c(“age_base”, “^age_change$”, “^DBP_base$”, “^WHR_base$”, “gender”, “icv”, “id”) to the data. Subject was defined as a random effect and we used the software’s default priors (i.e., JZS prior with a Cauchy prior on effect size and the Jeffreys prior on variance). We extracted Bayes Factors for the full model compared to models omitting the independent variables of interest. We calculated one-sided Bayes factors by drawing from the posterior distribution 10 times and calculating the probability of finding the effect in the expected direction. Then, we multiplied the two-sided Bayes Factor with this probability divided by 0.5 which represents equal likelihood of both directions (seeMorey (2017)for an example). We pooled the Bayes Factors by calculating the average and reported the range of obtained Bayes Factors from the five imputed datasets.

We interpreted a Bayes Factor between 3 and 6 as moderate evidence, and a Bayes Factor between 6 and 10 as positive evidence and above 10 as strong evidence in favor of the predictor. A Bayes Factor between 1/3 and 3 was deemed indecisive and a Bayes Factor smaller than 1/3 and 1/6 as moderate/strong evidence in favor of the null hypothesis. Bayes Factors were not corrected for multiple comparisons as they inherently provide a lower false positive rate.

Taken together, we rejected the null hypothesis if p < 0.033 and BF > 3. We accepted the null hypothesis if p > 0.033 and BF < 1/3 (seeTable 8).

#### 3.13.3 Transformations & checking of assumptions

All assumptions for LME were checked separately for the five imputed datasets (function test_LME_assumptions.R on github).

##### 3.13.3.1 Normality and homoscedasticity of residuals

We inspected the normality and homoscedasticity of residuals using qq-plots and plots of fitted versus residual values. Given the known skewness of WMH volumes, we transformed this measure using asinh-transformation as described above. The advantage of this transform is that it is also valid for zeros. We performed asinh-transformation of CESD for M2 and M3.

If for M1, the residuals were not normally distributed for all five imputed datasets, we implemented a generalized linear mixed model, using a Gamma error function and log link function instead of a linear mixed model. Here, we used the raw WMH volumes as outcome. We fit a robust linear mixed model (LMM) for all models M1–M3 to evaluate their robustness.

##### 3.13.3.2 Normality of random effects

We visually inspected the required normal distribution of the random effects.

##### 3.13.3.3 Influential cases

We used the function “influence” from the influence. ME package to assess influential cases. We plotted Cook’s distance for each model, and defined outliers as those cases with Cook’s distance > μ + 3 σ. We re-calculated all models without influential cases, and reported Bonferroni-corrected p-values of these models if they led to a different conclusion than the original models for any of the imputations.

##### 3.13.3.4 Model stability

We tested the stability of the linear mixed model with the command “glmm.model.stab” based on the code written by Roger Mundry. This function derives coefficients and their standard errors for all predictors while excluding levels of the random effects one at a time. If the function returned convergence issues, we tried to fix them by introducing a control object. Further, we inspected the summarized range of estimated coefficients and evaluated whether they differ substantially from the original coefficients.

##### 3.13.3.5 Variance inflation

We calculated variance inflation with the function “vif” from the car package omitting the random effect and interaction terms from the mixed models M1 - M4. A VIF above 10 was considered problematic and led to the inspection of a correlogram of all variables in the model. If two variables of interest were highly collinear, we calculated the residualized version of each of the predictors to infer its independent effect. If two control variables were highly collinear, we ignored their covariance.

#### 3.13.4 Exploratory analyses

*E1: Effects of baseline WHR and change in risk factors on WMH progression*

*Our power analysis revealed low power to detect the hypothesized effect size for the association with baseline WHR as well as change in blood pressure and WHR on WMH. There are very little data from longitudinal studies and our estimate was based on scaling of cross-sectional associations which might be biased and error-prone. We therefore tested these effects in exploratory analyses:*

- E1a: Higher WHR at baseline predicts a stronger increase of WMH volume at follow-up.
- E1b: Higher change in WHR predicts a stronger increase of WMH volume at follow-up.
- E1c: Higher change in DBP predicts a stronger increase of WMH volume at follow-up.

We used the statistical model M1

M1: asinh(WMH) ~ Age_baseline + Age_change + DBP_baseline + DBP_baseline:Age_change + DBP_change + WHR_baseline + WHR_baseline:Age_change + WHR_change + Gender + HT_medication + TIV + (1|subj)

and reported the effect size, p-value, and one-sided Bayes Factor for the interaction term of baseline WHR and age change, DBP change, and WHR change.

*E2: Gender-specific effects in WMH progression*

We did not perform power analyses for these hypotheses (E2a–E2c) due to missing reference values in the literature. Therefore, we explored whether WMH progression was more pronounced in women (E2a). We used a modified version of statistical model M1

M1E2a: asinh(WMH) ~ Age_baseline + Age_change + Gender + Gender:Age_change + DBP_baseline + DBP_baseline:Age_change + DBP_change + WHR_baseline + WHR_baseline:Age_change + WHR_change + HT_medication + TIV + (1|subj)

and reported the effect size, p-value, and one-sided Bayes Factor for the interaction term of gender and age change. We expected a positive coefficient for women.

We also explored whether there was an interactive effect of gender and DBP on WMH progression, where in women DBP has a stronger effect than in men (E2b). We used a modified version of statistical model M1

M1E2b: asinh(WMH) ~ Age_baseline + Age_change + Gender + Gender:Age_change + Gender:DBP_baseline + Gender:Age_change:DBP_baseline + DBP_baseline:Age_change + DBP_change + WHR_baseline + WHR_baseline:Age_change + WHR_change + HT_medication + TIV + (1|subj)

and reported the effect size, p-value, and one-sided Bayes Factor for the three-way interaction term of gender, age change, and DBP_baseline. We expected a positive coefficient for women.

We tested whether there was an interactive effect of gender and WHR on WMH progression, where in women WHR has a stronger effect than in men (E2c).

M1E2c: asinh(WMH) ~ Age_baseline + Age_change + Gender + Gender:Age_change + Gender:WHR_baseline + Gender:Age_change:WHR_baseline + DBP_baseline:Age_change + DBP_change + WHR_baseline + WHR_baseline:Age_change + WHR_change + HT_medication + TIV + (1|subj)

and reported the effect size, p-value, and one-sided Bayes Factor for the three-way interaction term of gender, age change, and WHR_baseline. We expected a positive coefficient for women.

*E3: Gender-specific effects of WMH progression on cognitive function*

Regarding cognitive function, we explored if there was an interactive effect of gender and WMH progression on executive cognitive function where in women WMH progression was associated with less decline in executive cognitive function (E3a). We used a modified model of M2

Z_exec ~ asinh(WMH)_baseline + WMH_change + Gender:WMH_change + Age_baseline + Age_change:asinh(WMH)_baseline + Age_change + Gender + education + CESD + (1|subj)

and reported the effect size, p-value, and one-sided Bayes Factor for the interaction term of gender and WMH change. We expected a positive coefficient for women.

Finally, we tested if there was an interactive effect of gender and WMH progression on global cognitive function where in women WMH progression was associated with less decline in global cognitive function (E3b).

We used a modified model of M3

Z_globalcog ~ asinh(WMH)_baseline + WMH_change + Gender:WMH_change + Age_baseline + Age_change:asinh(WMH)_baseline+ Age_change + Gender + education + CESD + (1|subj) and report the effect size, p-value, and one-sided Bayes Factor for the interaction term of gender and WMH change.

We expected a positive coefficient for women.

*Analyses of SBP and WMH progression*

We repeated model M1 using baseline SBP and change in SBP as predictor. We aimed to see whether changes in SBP would have a similar effect.

We performed the same imputation procedure as described above, using SBP instead of DBP.

*Progression of spatial patterns of WMH*

We performed an additional exploratory analysis to explore the spatial distribution of WMH progression.

Using the Bullseye approach, we divided the white matter into 36 parcels depending on their distance from the ventricles and the brain lobe and extracted regional WMH volumes (Sudre et al., 2018). We used each individual’s FreeSurfer longitudinal template to derive the WM lobar segmentation. The FreeSurfer commands mri_annotation2label and mri_aparc2aseg projected the cortical labels onto the WM.

In a second step, we calculated the distance from ventricles and cortical surfaces, and combined them to a normalized distance map from ventricle to cortex by dividing the distance to ventricle map by the sum of both.

We combined this map with the lobar segmentation, yielding 36 regions varying by distance to ventricles and brain region (seeFig. 1).

**Fig. 1.**
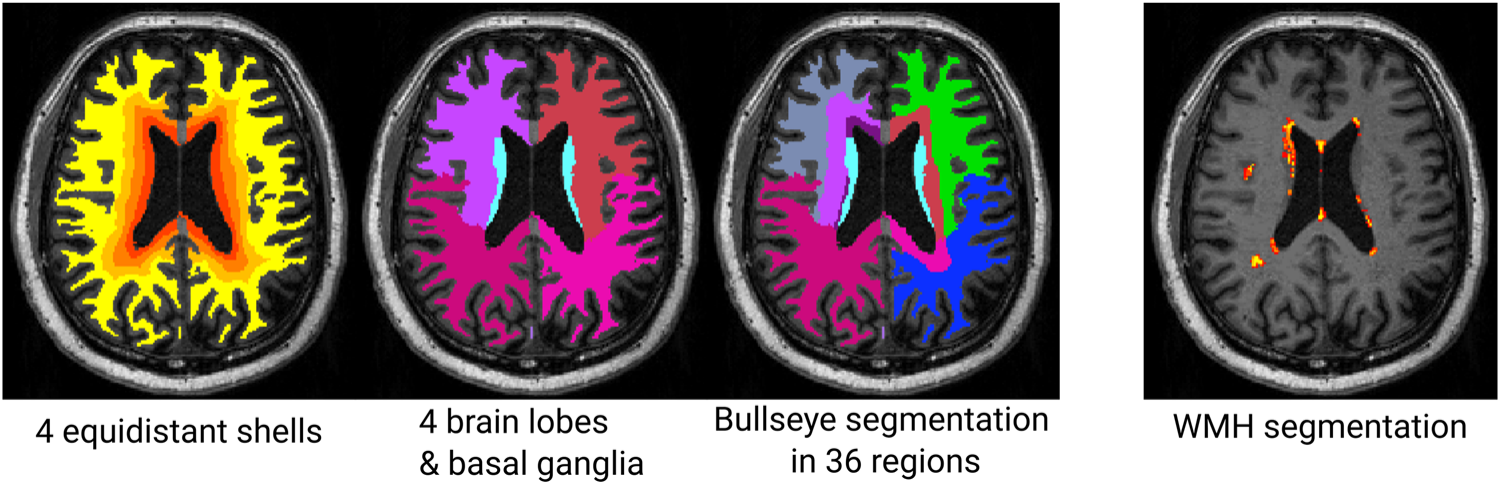
Example of Bullseye Segmentation. From left to right: Equidistant shells between ventricles and cortex, Segmentation of four brain lobes per hemisphere and basal ganglia region, combined Bullseye segmentation with 36 regions, probabilistic map of WMH.

For each participant this segmentation was co-registered to the FLAIR images from both time points using the registration matrix between the coregistered FLAIR and the longitudinal template T1. The registration was performed using bbregister.

WMH segmentation from longitudinal LST pipeline was binary in the longitudinally coregistered FLAIR space. After registering it to the longitudinal template, we thresholded the resulting values by 0.1 and extracted the sum of voxels in each of the 36 brain regions.

The Bullseye segmentation code was built on public repository and the complete pipeline is available on gitlab. WMH volumes in the bullseye parcels were adjusted by multiplying with the ratio of the individuals TIV to the mean TIV of the sample (Brugulat-Serrat et al., 2020). Following the approach byJiménez-Balado et al. (2022), we used principal component analysis (PCA) to extract WMH spatial components. We based the PCA on the baseline data and then projected the followup data on this decomposition using predict.psych. In the baseline data, we determined the optimal number of components using parallel analysis implemented in fa.parallel, resulting in 5 components.

We then used models M1–M3 with components as outcomes/predictors to assess the association of DBP/WHR and cognition with spatial WMH components.

## 4 Results

After applying exclusion criteria, the sample included 596 individuals (44.1 % females, mean age = 63.2 years) with two MRI assessments (seeFig. 2for details on the exclusion andTable 8for demographic characteristics at baseline).

**Fig. 2.**
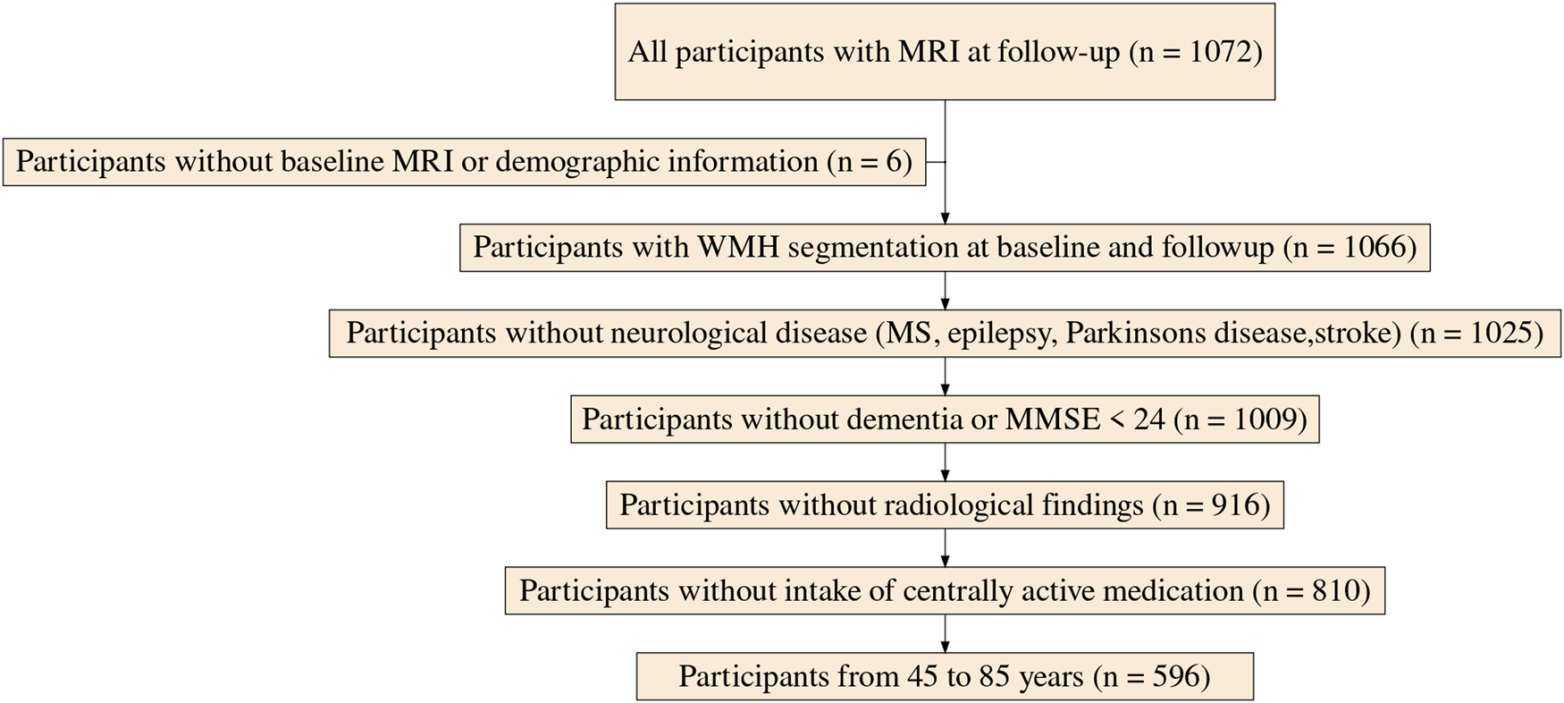
Flowchart of the study. MRI: magnetic resonance imaging, WMH: white matter hyperintensities, MS: multiple sclerosis, MMSE: mini-mental state examination.

### 4.1 Confirmatory analyses

**H1: Baseline DBP and WMH progression**

In model M1, we tested whether higher baseline DBP was associated with stronger WMH progression over time, independent of gender, age, and baseline and change in WHR. While higher age and DBP at baseline were associated with WMH volume cross-sectionally, there was no interaction of baseline DBP and time, speaking against effects of baseline DBP on progression of WMH (one-sided corrected p-value = 0.41 and BF = 0.04 with positive evidence against this hypothesis). The multivariate Wald test comparing a model with and without the interaction of baseline DBP and time, pooled across multiple imputations, yielded a p-value of 0.43.Table 9shows two-sided uncorrected p-values for all CVR factors.

**Table 9.** Association of baseline DBP with WMH progression.

	Estimate [95 % CI]	p-value
Age at baseline	0.056 [0.049, 0.062]	<0.001
Time	0.026 [-0.030, 0.083]	0.360
Baseline DBP	0.012 [0.005, 0.018]	<0.001
DBP change	0.006 [0.003, 0.009]	<0.001
Baseline WHR	0.843 [-0.117, 1.802]	0.085
WHR change	-0.084 [-0.682, 0.515]	0.784
DBP x time	0.000 [-0.001, 0.000]	0.612
WHR x time	0.029 [-0.017, 0.076]	0.218

Legend: Shown are unstandardized estimates with 95% confidence interval and p-value from linear mixed effect model M1. DBP: diastolic blood pressure, WHR: waist-to-hip ratio, WMH: white matter hyperintensities.

**Fig. 3.**
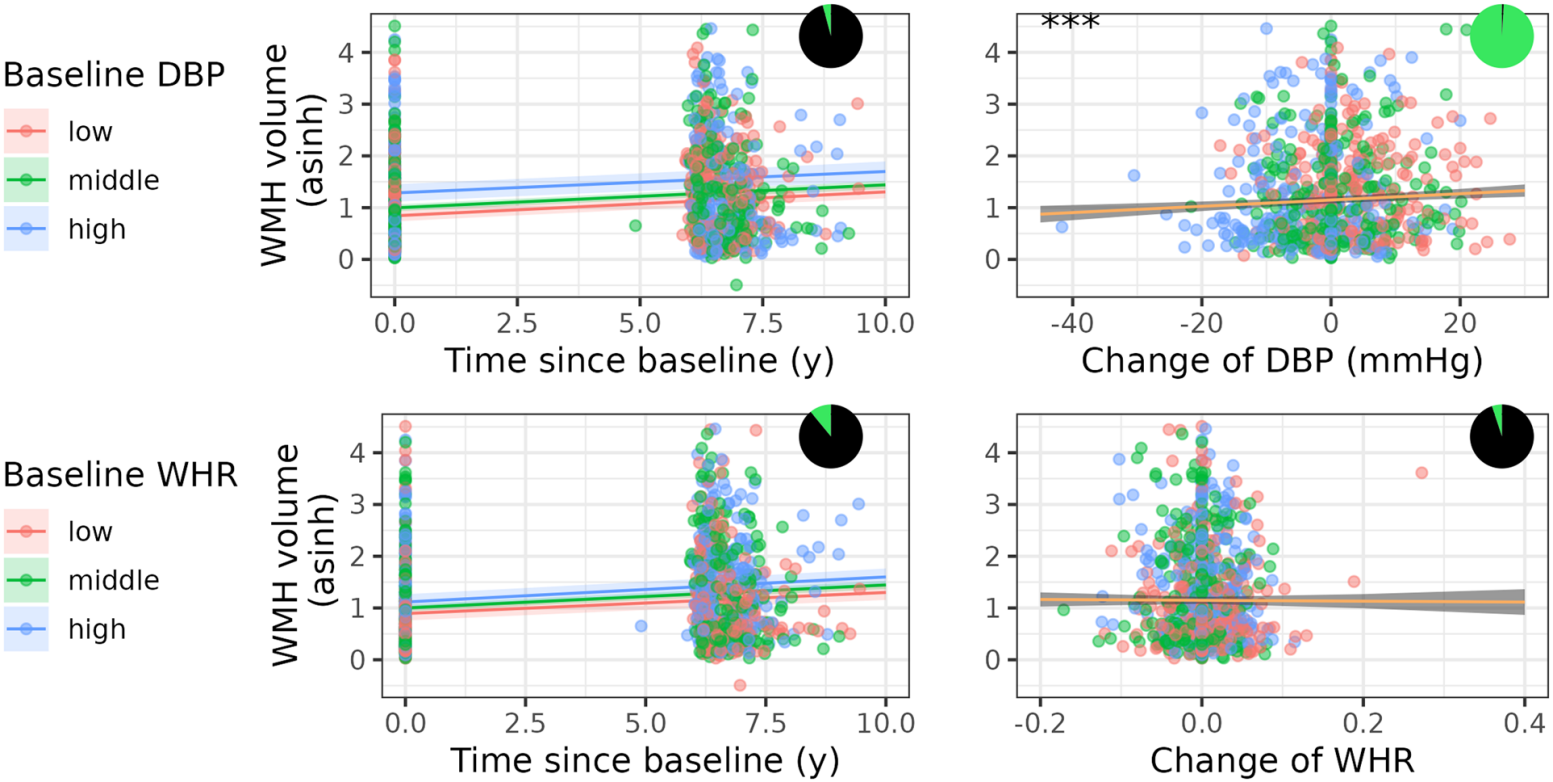
Association of baseline and change in DBP and WHR and WMH progression from model M1. Shown are model coefficients (with 95 % CI) for the interaction of baseline CVR risk and time (left side) and change in CVR (right side) on WMH volume on top of the real data. *** indicates p < 0.001. Red, green, and blue color indicates low, middle, and high baseline CVR. The pizza chart illustrates the Bayes Factor, for example, the likelihood in favor (green) and against (black) the alternative hypothesis. M1: statistical model 1, DBP: diastolic blood pressure, WHR: waist-hip ratio, WMH: white matter hyperintensities, CVR: cardiovascular risk.

Figure 3shows the associations of time by baseline CVR and change in CVR with WMH volume as well as Bayes Factor representations of the effects. For an illustration of age, DBP, and WMH volume in LIFE-Adult seeSupplementary Figures 6–7.

**H2: WMH progression and executive function**

In model M2, we investigated the association of higher WMH progression and executive function. There was no association of change in WMH volume with executive function (change in WMH volume: est(se) = −0.16(0.1)), corrected one-sided p-value = 0.08 and BF = 0.99 (seeTable 10). The multivariate Wald test comparing a model with and without the change in WMH volume, pooled across multiple imputations, yielded a p-value of 0.13. Based on the BF between 0.3 and 3, these results are inconclusive.

**Table 10.** Association of WMH progression and executive function.

	Estimate [95 % CI]	p-value
Age at baseline	-0.017 [-0.026, -0.008]	<0.001
Time	-0.047 [-0.061, -0.033]	<0.001
Baseline WMH volume	-0.017 [-0.116, 0.082]	0.740
Change in WMH volume	-0.159 [-0.360, 0.041]	0.120

Legend: Shown are unstandardized estimates with 95% confidence interval and p-value from linear mixed effect model M*2.*WMH: white matter hyperintensities.

**Fig. 4.**
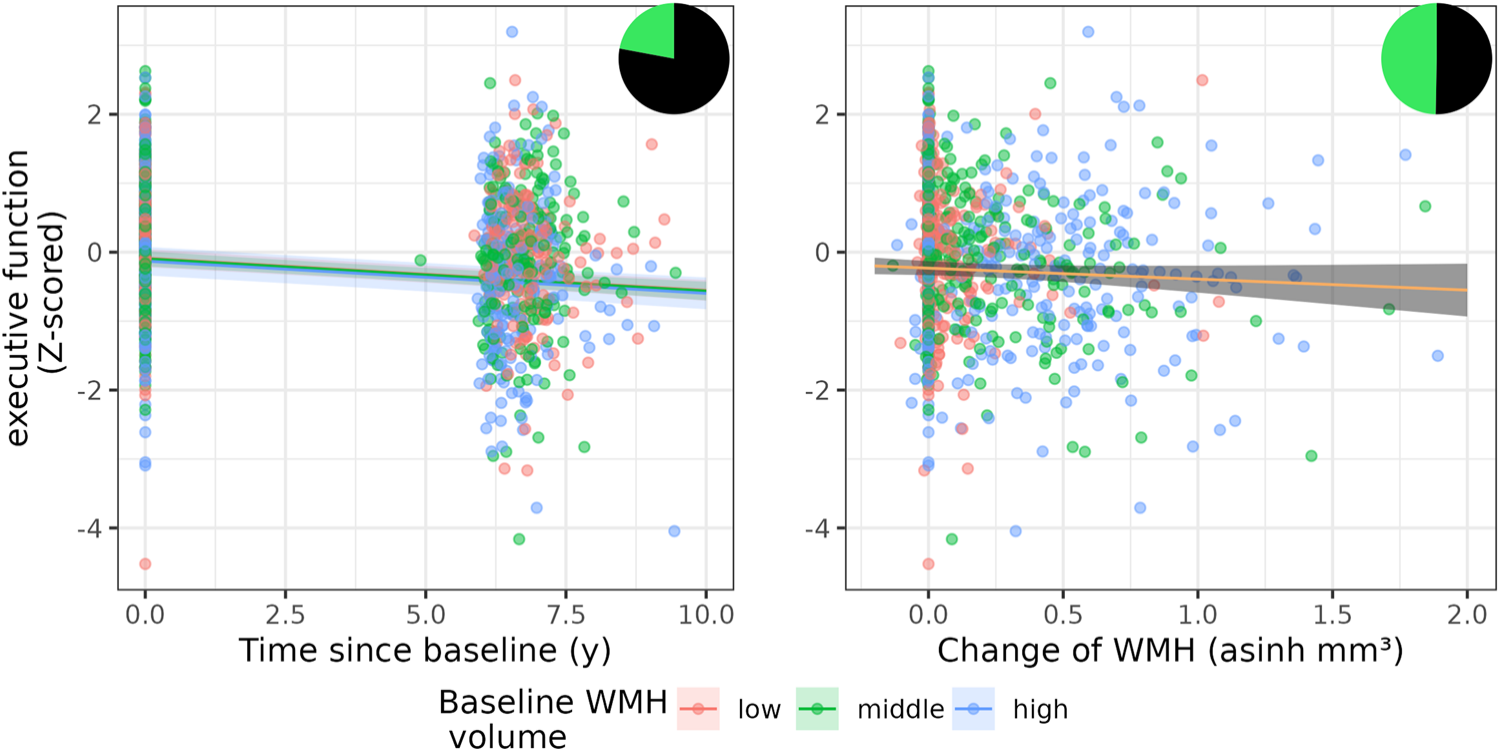
Association of baseline and change in asinh-transformed WMH volume and executive function from model M2. Shown are model coefficients (with 95 % CI) for the interaction of baseline WMH and time (left side) and change in WMH (right side) on executive function on top of the real data. Red, green, and blue color indicates low, middle, and high baseline WMH volume. The pizza chart illustrates the Bayes Factor, for example, the likelihood in favor (green) and against (black) the alternative hypothesis. M2: statistical model 2, WMH: white matter hyperintensities.

Figure 4shows the original data overlaid with the effect estimates from the model.

**H3: WMH progression and global cognitive function**

In model M3, we investigated the association of higher WMH progression and global cognition. Change in WMH volume was associated with change in global cognition, that is, increases in WMH related to decreases in cognition (est(se) = −0.33(0.09), corrected one-sided p-value = 0.0002 and BF = 153.38). The multivariate Wald test comparing a model with and without the change in WMH volume, pooled across multiple imputations, yielded a p-value of 0.01. This is interpreted as positive evidence for the alternative hypothesis.Table 11shows uncorrected two-sided p-values, andFigure 5shows the original data overlaid with the effect estimates from the model.

**Table 11.** Association of WMH progression and global cognitive function.

	Estimate [95 % CI]	p-value
Age at baseline	-0.045 [-0.053, -0.037]	<0.001
Time	-0.040 [-0.052, -0.028]	<0.001
Baseline WMH volume	-0.044 [-0.135, 0.047]	0.344
Change in WMH volume	-0.326 [-0.497, -0.154]	<0.001

Legend: Shown are unstandardized estimates with 95% confidence interval and p-value from linear mixed effect model M3. WMH: white matter hyperintensities.

**Fig. 5.**
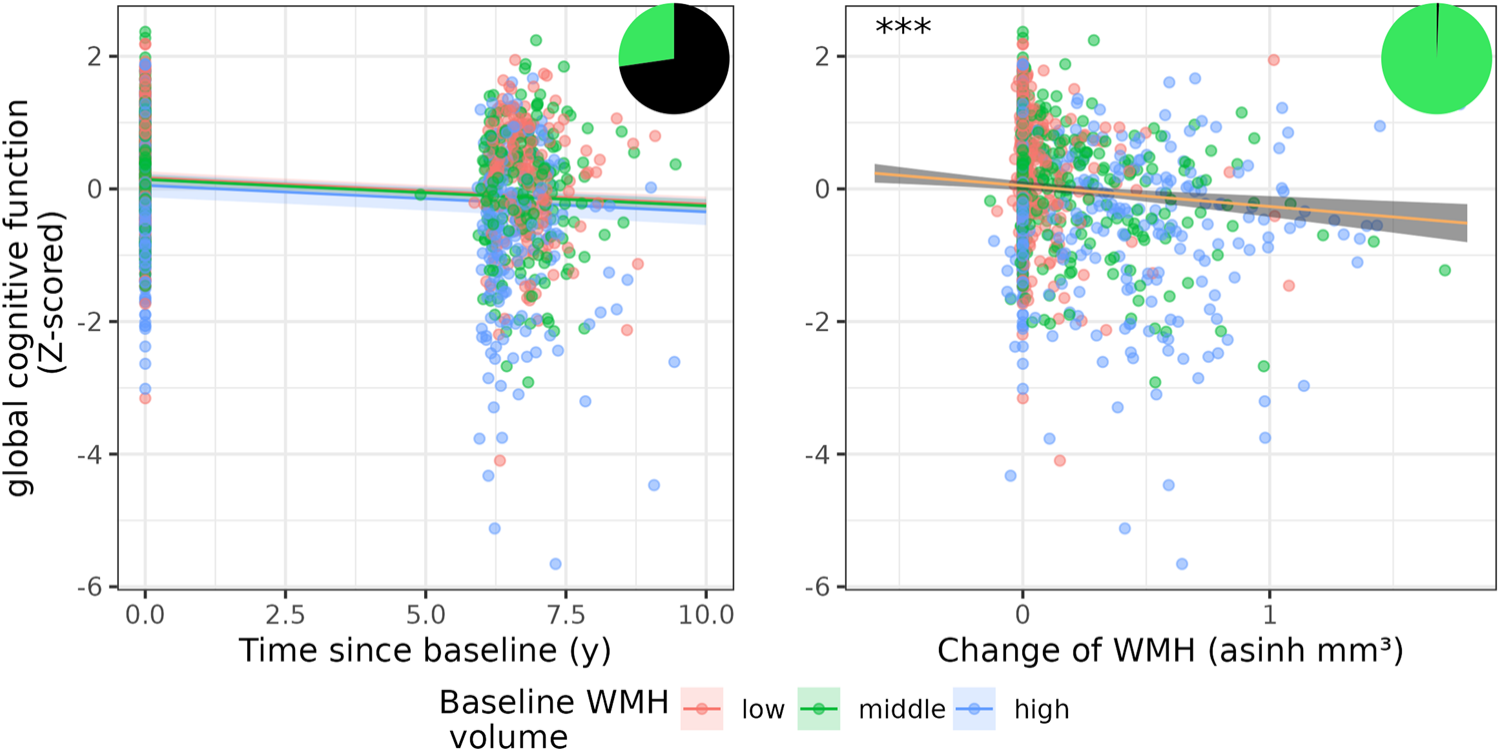
Association of baseline and change in asinh-transformed WMH volume and global cognitive function from model M3. Shown are model coefficients (with 95 % CI) for the interaction of baseline WMH and time (left side) and change in WMH (right side) on executive function on top of the real data. *** indicates p < 0.001. Red, green, and blue color indicates low, middle, and high baseline WMH volume. The pizza chart illustrates the Bayes Factor, for example, the likelihood in favor (green) and against (black) the alternative hypothesis. M3: statistical model 3, WMH: white matter hyperintensities.

### 4.2 Exploratory analyses

*E1a–E1c (DBP change, WHR baseline, WHR change)*

We hypothesized that higher WHR at baseline, and increases in WHR and in DBP would be associated with a stronger increase of WMH volume over time.

We used model 1 for exploring these associations. We found that a higher increase in DBP was associated with stronger progression of WMH volume (est(se) = 0.006 (0.002), p-value = 0.0003, and BF = 110.71). There were no significant associations for WHR change or the interaction of age and WHR (seeFig. 2andTable 9).

*E2a–E3b: Interactions of gender, CVR risk, WMH progression, and cognition*

While men had significantly lower WMH volume at baseline (est(se) = −0.28 (0.09), p-value = 0.002), there was no significant interaction of gender and WMH progression, gender and DBP change on WMH progression or any interaction with DBP or WHR at baseline and WHR change.

Men performed worse in executive function (est(se) = −0.24 (0.08), p-value = 0.002) and global cognitive function (est(se) = −0.3 (0.07), p-value = 0.00006) than women.

There was no significant interaction of gender and change in WMH volume on executive function or global cognitive function change (seeSupplementary Tables 2–7andTables 12,13, and14for gender-stratified results).

**Table 12.** Gender-stratified results for model M1 on CVR factors.

	Females: estimate[95 % CI]	Females:p-value	Males: estimate[95 % CI]	Males:p-value
Age at baseline	0.058 [0.046, 0.069]	<0.001	0.054 [0.045, 0.063]	<0.001
Time	0.019 [-0.085, 0.124]	0.716	0.032 [-0.071, 0.135]	0.541
Baseline DBP	0.016 [0.005, 0.026]	0.003	0.009 [0.000, 0.017]	0.044
DBP change	0.009 [0.004, 0.014]	<0.001	0.004 [0.000, 0.008]	0.076
Baseline WHR	0.671 [-0.867, 2.210]	0.392	0.945 [-0.307, 2.197]	0.139
WHR change	0.132 [-0.685, 0.949]	0.751	-0.472 [-1.424, 0.481]	0.331
DBP x time	0.000 [-0.001, 0.001]	0.653	0.000 [-0.001, 0.000]	0.234
WHR x time	0.013 [-0.091, 0.116]	0.808	0.046 [-0.042, 0.134]	0.304

Legend: Shown are unstandardized estimates with 95% confidence interval and p-value from linear mixed effect model M1, stratified by gender. DBP: diastolic blood pressure, WHR: waist-to-hip ratio,WMH: white matter hyperintensities.

**Table 13.** Gender-stratified results for model M2 on executive function.

	Females: estimate[95 % CI]	Females:p-value	Males: estimate[95 % CI]	Males:p-value
Age at baseline	-0.019 [-0.032, -0.007]	0.003	-0.015 [-0.027, -0.003]	0.016
Time	-0.037 [-0.058, -0.015]	<0.001	-0.055 [-0.075, -0.036]	<0.001
Baseline WMH volume	0.096 [-0.037, 0.229]	0.155	-0.127 [-0.273, 0.019]	0.087
Change in WMH volume	-0.208 [-0.517, 0.100]	0.185	-0.111 [-0.375, 0.154]	0.413

Legend: Shown are unstandardized estimates with 95% confidence interval and p-value from linear mixed effect model M2, stratified by gender. WMH: white matter hyperintensities.

**Table 14.** Gender-stratified results for model M3 on global cognitive function.

	Females: estimate[95 % CI]	Females:p-value	Males: estimate[95 % CI]	Males:p-value
Age at baseline	-0.040 [-0.053, -0.028]	<0.001	-0.048 [-0.059, -0.037]	<0.001
Time	-0.028 [-0.046, -0.010]	0.003	-0.050 [-0.067, -0.034]	<0.001
WMH volume baseline	0.022 [-0.104, 0.149]	0.728	-0.113 [-0.244, 0.017]	0.089
Change in WMH volume	-0.371 [-0.636, -0.107]	0.006	-0.284 [-0.510, -0.058]	0.014

Legend: Shown are unstandardized estimates with 95% confidence interval and p-value from linear mixed effect model M3, stratified by gender. WMH: white matter hyperintensities.

*E4: Association of SBP and WMH progression*

We also explored the association of SBP at baseline, change of SBP, and WMH progression and found that baseline SBP predicted WMH progression (est(se) = 0.00046(0.002), p-value = 0.002, Wald p-value = 0.03, seeTable 15). Similar to DBP change, SBP increase was also associated with WMH progression (est(se) = 0.005(0.002), p-value = 6*10^-9^).

**Table 15.** Results of an exploratory model testing the association of baseline and change in SBP with WMH progression.

	Estimate [95 % CI]	p-value
Age at baseline	0.0504 [0.0438, 0.0571]	<1e-04
Time between baseline and followup	-0.0331 [-0.0870, 0.0208]	0.2290
Systolic BP at baseline	0.0051 [0.0013, 0.0089]	0.0082
Change in Systolic BP	0.0050 [0.0033, 0.0067]	<1e-04
Waist-to-hip ratio at baseline	0.9273 [-0.0327, 1.8872]	0.0583
Change in WHR	-0.1219 [-0.7154, 0.4717]	0.6871
Interaction of time and SBP at baseline	0.0005 [0.0002, 0.0007]	0.0017
Interaction of time and WHR at baseline	0.0148 [-0.0318, 0.0613]	0.5338

Legend: Shown are unstandardized estimates with 95% confidence interval and p-value from linear mixed effect model M1 using SBP as predictor. SBP: systolic blood pressure, WHR: waist-to-hip ratio, WMH: white matter hyperintensities.

*E5: Spatial patterns of WMH progression*

We found that WMH progression was most pronounced in the frontal and parietal WM, especially in shells closer to the ventricles (seeFig. 6A).

**Fig. 6.**
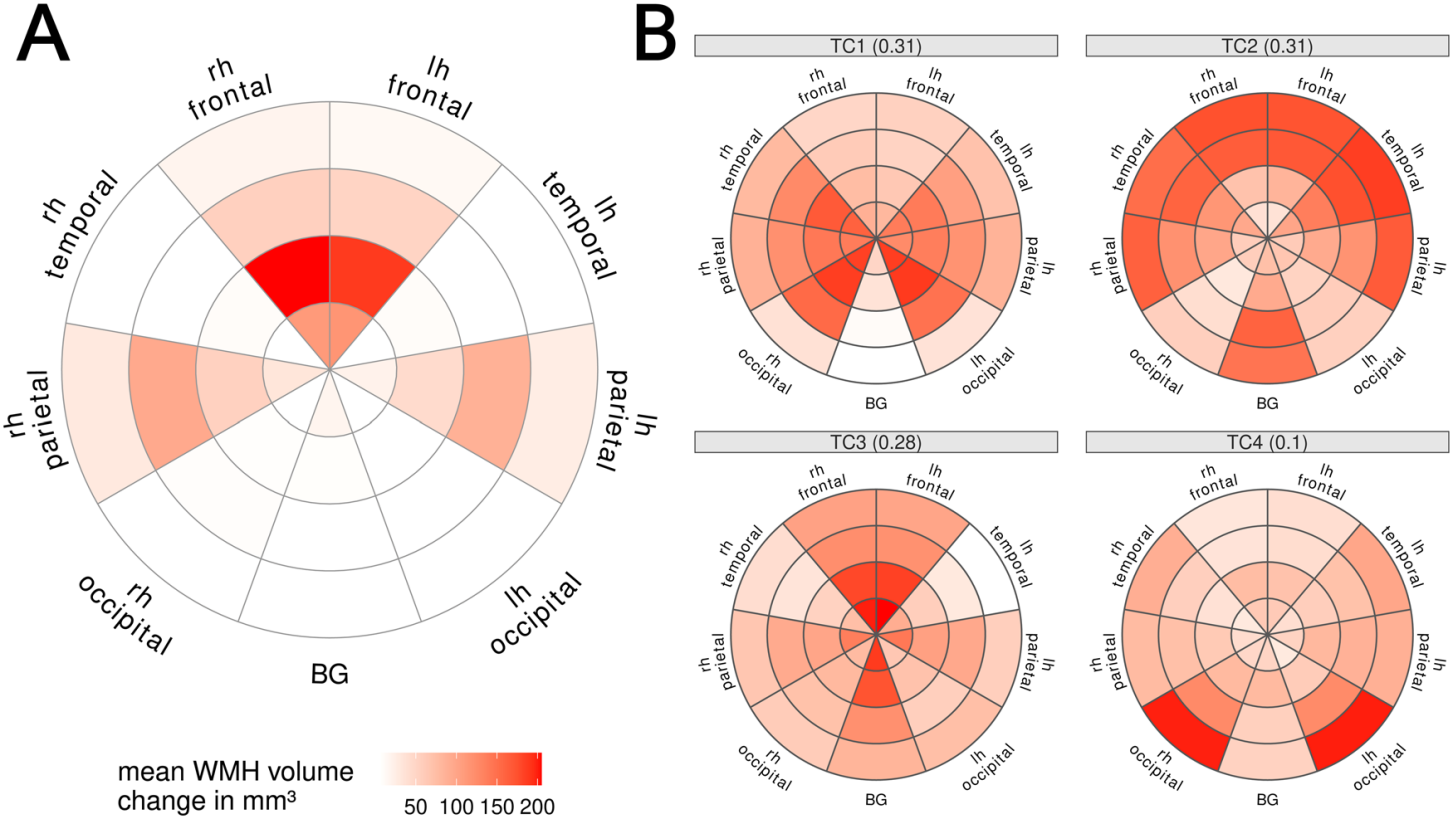
(A) Average white matter hyperintensities (WMH) volume increase between baseline and follow-up in Bullseye WM regions. Distance from center represents four equidistant shells between ventricles and cortex, and angular orientation represents cerebral lobes and hemisphere. (B) WMH spatial pattern derived from baseline WMH distribution. Labels indicate component (TC) number and variance (in %) explained. Distance from center represents four equidistant shells between ventricles and cortex, and angular orientation represents cerebral lobes and hemisphere. Colors indicate contribution of this region to the pattern. lh: left hemisphere, rh: right hemisphere, BG: basal ganglia

Parallel analysis indicated four to be the optimal number of spatial WMH components (shown inFig. 6B). Component 1 included occipital periventricular WMH; component 2 was composed of WMH in frontal, temporal, and parietal deeper WM as well as basal ganglia. Component 3 included frontal and basal ganglia periventricular WMH, and component 4 mainly included deep occipital WMH.

When investigating the associations of DBP and WHR with the four components, we found that all components were cross-sectionally associated with age. Yet, longitudinally, progression only occurred in component 3. Here, both time (est(SD) = 0.0057(0.02), p-value = 0.005) and change in DBP (est(SD) = 0.0045 (0.0012), p-value = 0.00015) were independently associated with higher scores (seeTable 16).

**Table 16.** Associations of DBP and WHR with progression of spatial WMH components.

	C1: estimate[95 % CI]	C1: p-value	C2: estimate[95 % CI]	C2:p-value	C3: estimate[95 % CI]	C3:p-value	C4: estimate[95 % CI]	C4:p-value
Age at baseline	0.061 [0.053, 0.069]	<0.001	0.053 [0.044, 0.062]	<0.001	0.064 [0.055, 0.072]	<0.001	0.035 [0.025, 0.044]	<0.001
Time	0.027 [-0.008, 0.062]	0.123	0.005 [-0.021, 0.030]	0.729	0.057 [0.018, 0.097]	0.005	-0.011 [-0.034, 0.011]	0.332
Baseline DBP	0.002 [-0.006, 0.010]	0.611	0.010 [0.001, 0.018]	0.024	0.009 [0.001, 0.017]	0.027	0.006 [-0.003, 0.015]	0.174
DBP change	0.001 [-0.001, 0.003]	0.290	0.001 [0.000, 0.003]	0.117	0.004 [0.002, 0.007]	<0.001	0.001 [0.000, 0.002]	0.133
Baseline WHR	0.415 [-0.487, 1.317]	0.367	-0.478 [-1.457, 0.501]	0.338	-0.085 [-1.014, 0.843]	0.857	0.280 [-0.734, 1.294]	0.588
WHR change	-0.220 [-0.598, 0.158]	0.254	0.010 [-0.264, 0.284]	0.945	-0.095 [-0.518, 0.329]	0.661	0.113 [-0.132, 0.357]	0.366
DBP x time	0.000 [0.000, 0.000]	0.971	0.000 [0.000, 0.000]	0.531	0.000 [0.000, 0.000]	0.597	0.000 [0.000, 0.000]	0.348
WHR x time	-0.018 [-0.047, 0.011]	0.213	-0.001 [-0.022, 0.020]	0.919	-0.020 [-0.053, 0.012]	0.224	0.007 [-0.012, 0.026]	0.449

Legend: Shown are unstandardized estimates with 95% confidence interval and p-values from linear mixed effect model M1. C1-C4: spatial WMH components, DBP: systolic blood pressure, WHR: waist-to-hip ratio, WMH: white matter hyperintensities.

We found that only increases in C3 were significantly associated with increased decline in global cognitive function (est(SD) = −0.074(0.035), p = 0.034) (seeTable 17). None of the components was associated with decline in executive function.

**Table 17.** Associations of WMH spatial component progression with global cognitive function.

	C1: estimate[95 % CI]	C1: p-value	C2: estimate[95 % CI]	C2: p-value	C3: estimate[95 % CI]	C3: p-value	C4: estimate[95 % CI]	C4: p-value
Age at baseline	-0.049 [-0.058, -0.041]	<0.001	-0.051 [-0.058, -0.043]	<0.001	-0.049 [-0.057, -0.041]	<0.001	-0.052 [-0.059, -0.045]	<0.001
Time	-0.054 [-0.064, -0.044]	<0.001	-0.054 [-0.064, -0.045]	<0.001	-0.053 [-0.063, -0.043]	<0.001	-0.054 [-0.064, -0.045]	<0.001
Baseline component score	-0.036 [-0.109, 0.038]	0.338	0.001 [-0.068, 0.069]	0.985	-0.030 [-0.101, 0.041]	0.412	0.024 [-0.042, 0.090]	0.483
Change in component score	-0.048 [-0.119, 0.023]	0.186	-0.058 [-0.124, 0.008]	0.086	-0.074 [-0.143, -0.006]	0.034	-0.010 [-0.075, 0.055]	0.765

Legend: Shown are unstandardized estimates with 95% confidence interval and p-values from linear mixed effect model M3. C1-C4: spatial WMH components, WMH: white matter hyperintensities.

### 4.3 Model assumptions

Residuals and random effects were normally distributed for all models (seeSupplementary Figures 1, 4 and 5). All VIF were below 10.

Estimates for the predictors of interest of models M1, M2, and M3 are depicted inFigure 7. Each figure shows original estimates with minimal and maximal estimates from random effect stability tests across imputations, robust LMM estimates with 95% CI across imputations, and estimates with 95% CI from models without influential cases for each imputed dataset. There were 7, 27, and 20 unique influential cases across the five imputations for model M1, M2, and M3, respectively.

**Fig. 7.**
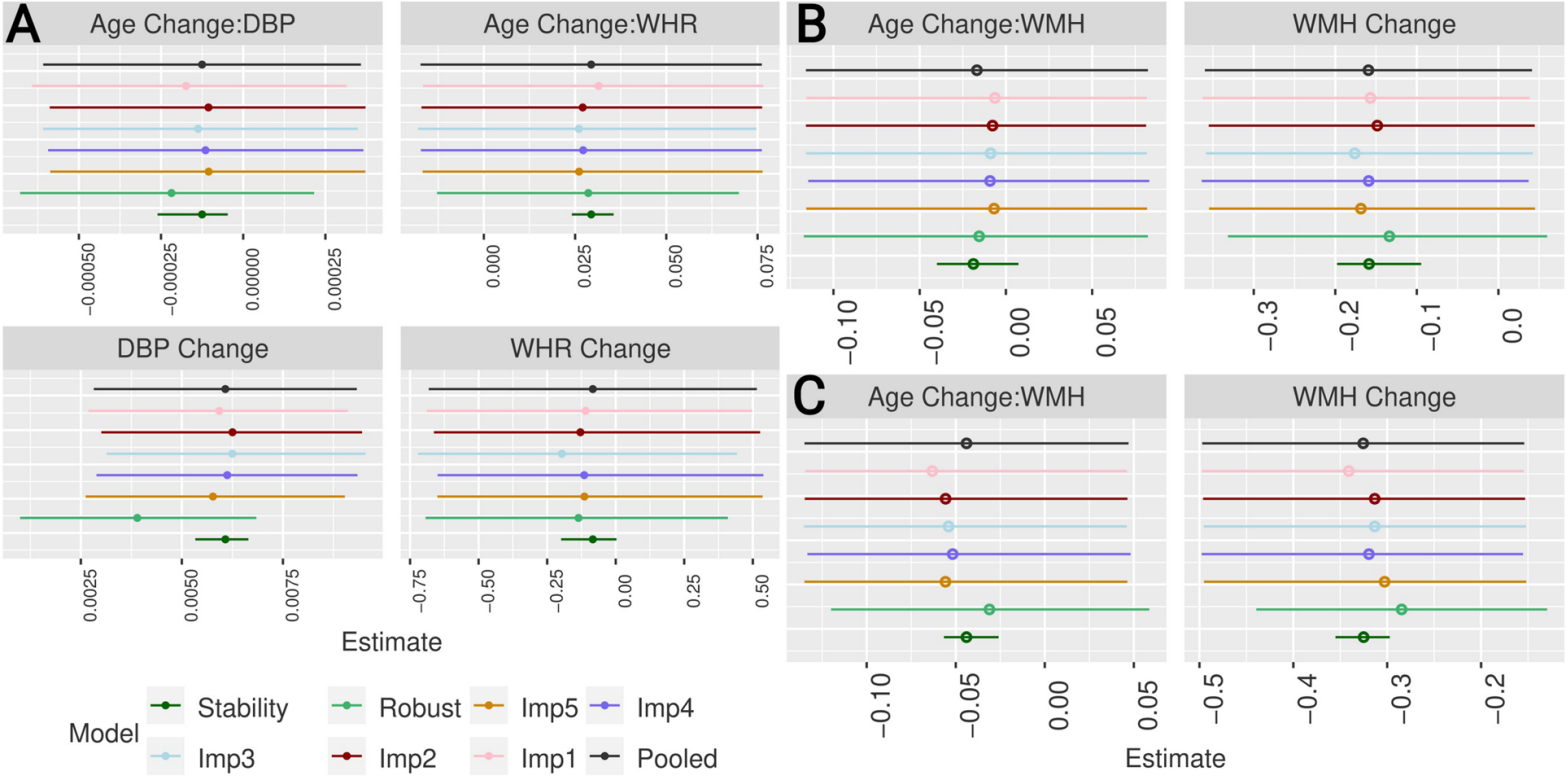
Original pooled estimate (dark grey) and 95% CI, minimum and maximum estimate derived from random effect stability analysis (dark green), estimate and 95% CI from robust LMM (light green), and estimates and 95% CI from individual imputations without influential cases for each imputed dataset. For (A): M1 (Change in WMH volume) (B): M2 (Executive function) (C): M3 (Global cognitive function)

In M1, the estimate for the interaction of baseline DBP and time (H1) as well as the estimates for DBP change (E1a) and WHR change (E1c) agreed well between the original model and models without influential cases, individual random effects and robust LMM. Only estimates for WHR change (E1b) came with high uncertainty and should be interpreted carefully.

In Model M2, influential cases biased the estimates towards higher values for WMH change. Robust estimates showed a similar trend.

In Model M3, this bias for WMH change was smaller. Still, the robust estimates were somewhat smaller than the original ones.

## 5 Discussion

In this registered report, we studied the progression of WMH with respect to risk factors and cognitive outcomes. We pre-registered three confirmatory hypotheses of which one was accepted. We found a significant association of WMH progression and global cognitive decline over 6 years. There was no significant effect of baseline DBP on WMH progression nor an association of WMH progression and executive function decline, with Bayes Factors indicating inconclusive results.

In exploratory analyses, we found that increase in DBP was a significant predictor of WMH progression, independent of baseline DBP. WHR at baseline or change in WHR did not predict WMH progression. SBP at baseline and increase of SBP also predicted WMH progression. There was no significant interaction with gender for any of the effects. Descriptively, women had higher WMH volume than men and performed better in executive function and global cognitive function at baseline. WMH progression was mostly located in frontal periventricular regions, where also effects of blood pressure were most pronounced.

The mean annual WMH progression in this study was 0.17 cm^3^/y. This was comparable to the estimation in our power analysis (0.32 cm^3^/y) but lower than the figure reported byBrown et al. (2021)(0.7 cm^3^/y) who also included stroke and dementia patients. Against our hypotheses, we did not find an interaction effect of baseline DBP with time since baseline on the progression of WMH. Previous studies mostly investigated annual change in WMH volume as outcome in a linear model, while we took the more flexible approach of using a mixed model. Both approaches should yield equivalent results (Chapter 17,Walker, 2018).

In additional analyses, we found stronger effects of both baseline and change in DBP when using annual change in WMH volume as outcome in a linear model like inDebette et al. (2011)(both p < 10^-5^, seeSupplementary Table 1,Supplementary Figs. 1-3). However, these models did not satisfy the assumptions of the linear model (non-normal distribution of residuals), possibly due to the zero-inflated and left-bounded distribution of WMH change (seesupplementary Fig. 2). In a model using change based on asinh-transformed WMH volumes at baseline and follow-up as outcome, the association of baseline DBP with WMH change was also present while the residuals indicated a bad model fit (seeSupplementary Fig. 3). Taken together, our findings imply a considerable effect of the transformation of WMH volume and the selected statistical approach on regression estimates, underlining the importance of assumption verification and transparent reporting.

In our confirmatory analysis, we found higher DBP increase related to increase in WMH volume, independent of baseline DBP. This was seen in both the preregistered mixed model analysis and the change score models. Additionally, in an exploratory analyses, we found that higher SBP at baseline was associated with WMH progression in line with the literature (Brown et al., 2021). We also found that increases in SBP were associated with WMH progression, a finding that has to our knowledge only been described in one study (Godin et al., 2011) but is in line with the protective effects of intensive blood pressure control (Lai et al., 2020). This was seen in both the preregistered mixed model analysis and in the change score models.

DBP reflects the balance between peripheral vascular resistance and large artery stiffness while SBP increases with both vascular resistance and large artery stiffness (Pinto, 2007). In the course of aging, SBP and DBP increase in parallel, driven by both vascular resistance and large arterial stiffness until around 55 years. After that, large artery stiffness dominates and leads to further increases of SBP while DBP levels off or slightly decreases (Franklin et al., 1997;Kaess et al., 2012). Previous studies have stressed the stronger association of concurrent SBP with WMH volume in the elderly, and a stronger effect of mid-life DBP on WMH in late-life (Wartolowska & Webb, 2021). While this was a cross-sectional study, our results supported the stronger effect of baseline SBP compared to baseline DBP but similar effects of BP change (Wilkinson & Webb, 2022).

Previous studies have focused on SBP reduction due to its strong age-related increase and greater importance for cardiovascular events in the elderly (Wang et al., 2005). In the SPRINT-MIND trial, the intensive control of SBP group (mean after intervention of 120 mmHg) versus the standard SBP control group (mean of 135 mmHg) showed significantly less WMH progression (0.92 cm^3^vs. 1.45 cm^3^) (Nasrallah et al., 2019). DBP also reduced in the main SPRINT trial but no data were reported in relation to WMH progression (SPRINT Research Group et al., 2015). Intensive BP control did not induce hypoperfusion inCroall et al. (2018)but excessively low DBP might be associated with an increased risk for stroke and cardiovascular disease (Somes et al., 1999).

We did not find evidence for an association of abdominal obesity with WMH progression. Despite obesity being a risk factor for dementia, its association with imaging markers of cSVD is relatively small compared to hypertension (Arnoldussen et al., 2019;Dearborn et al., 2015;Debette et al., 2011;Livingston et al., 2020). As also indicated by the power analysis, the effect size could have been too small to be detected with this sample size. We found a strong correlation of 0.75 between WHR and MRI-based visceral fat estimation in a subset of 251 individuals from the study sample which leads us to believe that our WHR measure was an adequate reflection of abdominal obesity.

In line with previous studies, we found that WMH progression was associated with global cognitive decline (Hamilton, Cox, et al., 2021;Kloppenborg et al., 2014). This association amounted to a reduction of -0.029 in the normalized global cognition score per 1 cm^3^of WMH volume increase (while adjusting for age and time elapsed). A year elapsed accounted for -0.048 decrease in global cognition, or -0.043 when simultaneously adjusting for WMH change.

We did not find any gender-specific associations of risk factors or cognitive outcomes with WMH progression. While females had higher WMH volumes at baseline, progression was similar in line with previous studies (Brown et al., 2021;Lohner et al., 2022). We did not find evidence for a differential association of cardiovascular risk factors and WMH between genders as suggested inAlqarni et al. (2021),Sachdev et al. (2009),Bonberg et al. (2022), andSchindler et al. (2023). This might be due to the age range of our sample which included pre-, peri-, and post-menopausal women. This might have reduced the power to detect differential effects which might be most pronounced in the transition phase (Schindler et al., 2023). Also regarding the cognitive consequences of WMH progression, no differences between genders were seen. Still, we cannot rule out that we were underpowered to detect these effects or that gender differences might have been observed in studies including more pre-and peri-menopausal women. Finally, in an exploratory analysis of spatial patterns of WMH, we found that WMH progression mainly occurred in the frontal periventricular WM. Progression was also observed in occipital periventricular WM but less so in deep occipital WM. This is in line with previous reports of gradual extension of existing WMH (which start to appear around the ventricles) into nearby normal appearing WM (Promjunyakul et al., 2018). Stronger DBP increase was also associated with frontal-periventricular WMH progression. There was no DBP or WHR-associated increase in any other spatial patterns. Specifically, component 4, including deep occipital WMH which might be reflective of cerebral amyloid angiopathy, did not change in relation to the risk factors and was overall relatively stable (Charidimou et al., 2022;Thanprasertsuk et al., 2014). The lack of more specific associations might also reflect the relatively low total WMH volume and progression and limited variability in the spatial distribution of WMH in this cohort of community-dwelling non-demented elderly.

## 6 Strengths and Limitations

Strengths of this study are the rigorously preregistered hypotheses and openly accessible analyses in a well-powered and deeply phenotyped dataset. Limitations of the study are the lack of power for exploring gender effects, the lack of information on menopausal status, and the WEIRDness (Western Educated Industrialized Rich Democracies) of the sample. Results should be replicated in a more diverse sample to ensure generalizability (Kawas et al., 2021)

## 7 Conclusion

Taken together, these results indicate that strict control of blood pressure in both men and women would contribute to limit WMH progression and related global cognitive decline. Further research is needed, especially well-powered longitudinal assessments of WMH progression across mid-age and menopause, in order to establish sex/gender-specific guidelines on optimal vascular risk management for brain health.

## Supplementary Material

Supplementary Material

## Data Availability

Due to potential identifiability of individuals from demographic and medical information, we shared a surrogate version of the dataset onhttps://github.com/fBeyer89/VRF-and-progression-of-WMHalong with the code for data analysis and power calculation (Nowok et al., 2016). Raw data of the LIFE-Adult cohort can be requested via the LIFE study center (https://ldp.life.uni-leipzig.de/).

## Footnotes

1 The term “white matter lesions” (WMH) was replaced by white matter hyperintensities (WMH) throughout the manuscript according toDuering et al. (2023)

## Supplementary Materials

Supplementary material for this article is available here:https://osf.io/dynfc

## References

[b1] Alqarni , A. , Jiang , J. , Crawford , J. D. , Koch , F. , Brodaty , H. , Sachdev , P. , & Wen , W. ( 2021 ). Sex differences in risk factors for white matter hyperintensities in non-demented older individuals . Neurobiology of Aging , 98 , 197 – 204 . 10.1016/j.neurobiolaging.2020.11.001 33307330

[b2] Armstrong , N. J. , Mather , K. A. , Sargurupremraj , M. , Knol , M. J. , Malik , R. , Satizabal , C. L. , Yanek , L. R. , Wen , W. , Gudnason , V. G. , Dueker , N. D. , Elliott , L. T. , Hofer , E. , Bis , J. , Jahanshad , N. , Li , S. , Logue , M. A. , Luciano , M. , Scholz , M. , Smith , A. V. , … Nyquist , P. A. ( 2020 ). Common genetic variation indicates separate causes for periventricular and deep white matter hyperintensities . Stroke , 51 ( 7 ), 2111 – 2121 . 10.1161/STROKEAHA.119.027544 32517579 PMC7365038

[b3] Arnoldussen , I. A. C. , Gustafson , D. R. , Leijsen , E. M. C. , de Leeuw , F.-E. , & Kiliaan , A. J. ( 2019 ). Adiposity is related to cerebrovascular and brain volumetry outcomes in the RUN DMC study . Neurology , 93 ( 9 ), e864 . 10.1212/WNL.0000000000008002 31363056

[b4] Beyer , F. , Kharabian Masouleh , S. , Huntenburg , J. M. , Lampe , L. , Luck , T. , Riedel-Heller , S. G. , Loeffler , M. , Schroeter , M. L. , Stumvoll , M. , Villringer , A. , & Witte , A. V. ( 2017 ). Higher body mass index is associated with reduced posterior default mode connectivity in older adults . Human Brain Mapping , 38 ( 7 ), 3502 – 3515 . 10.1002/hbm.23605 28397392 PMC6867016

[b5] Bonberg , N. , Wulms , N. , Dehghan-Nayyeri , M. , Berger , K. , & Minnerup , H. ( 2022 ). Sex-specific causes and consequences of white matter damage in a middle-aged cohort . Frontiers in Aging Neuroscience , 14 , 810296 . 10.3389/fnagi.2022.810296 35645786 PMC9131069

[b6] Bos , D. , Wolters , F. J. , Darweesh , S. K. L. , Vernooij , M. W. , de Wolf , F. , Ikram , M. A. , & Hofman , A. ( 2018 ). Cerebral small vessel disease and the risk of dementia: A systematic review and meta-analysis of population-based evidence . Alzheimer’s & Dementia , 14 ( 11 ), 1482 – 1492 . 10.1016/j.jalz.2018.04.007 29792871

[b7] Brown , R. , Low , A. , & Markus , H. S. ( 2021 ). Rate of, and risk factors for, white matter hyperintensity growth: A systematic review and meta-analysis with implications for clinical trial design . Journal of Neurology, Neurosurgery & Psychiatry , 92 ( 12 ), 1271 – 1277 . 10.1136/jnnp-2021-326569 34344790

[b8] Brugulat-Serrat , A. , Salvadó , G. , Sudre , C. H. , Grau-Rivera , O. , Suárez-Calvet , M. , Falcon , C. , Sánchez-Benavides , G. , Gramunt , N. , Fauria , K. , Cardoso , M. J. , Barkhof , F. , Molinuevo , J. L. , Gispert , J. D. , Camí , J. , Cacciaglia , R. , Operto , G. , Skouras , S. , Minguillón , C. , Polo , A. , … for the ALFA Study . ( 2020 ). Patterns of white matter hyperintensities associated with cognition in middle-aged cognitively healthy individuals . Brain Imaging and Behavior , 14 ( 5 ), 2012 – 2023 . 10.1007/s11682-019-00151-2 31278650 PMC7572336

[b9] Charidimou , A. , Boulouis , G. , Frosch , M. P. , Baron , J.-C. , Pasi , M. , Albucher , J. F. , Banerjee , G. , Barbato , C. , Bonneville , F. , Brandner , S. , Calviere , L. , Caparros , F. , Casolla , B. , Cordonnier , C. , Delisle , M.-B. , Deramecourt , V. , Dichgans , M. , Gokcal , E. , Herms , J. , … Greenberg , S. M. ( 2022 ). The Boston criteria version 2.0 for cerebral amyloid angiopathy: A multicentre, retrospective, MRI-neuropathology diagnostic accuracy study . The Lancet Neurology , 21 ( 8 ), 714 – 725 . 10.1016/S1474-4422(22)00208-3 35841910 PMC9389452

[b10] Croall , I. D. , Tozer , D. J. , Moynihan , B. , Khan , U. , O’Brien , J. T. , Morris , R. G. , Cambridge , V. C. , Barrick , T. R. , Blamire , A. M. , Ford , G. A. , Markus , H. S. , & for the PRESERVE Study Team . ( 2018 ). Effect of standard vs intensive blood pressure control on cerebral blood flow in small vessel disease: The PRESERVE randomized clinical trial . JAMA Neurology , 75 ( 6 ), 720 – 727 . 10.1001/jamaneurol.2017.5153 29507944 PMC5885221

[b11] d’Arbeloff , T. , Elliott , M. L. , Knodt , A. R. , Melzer , T. R. , Keenan , R. , Ireland , D. , Ramrakha , S. , Poulton , R. , Anderson , T. , Caspi , A. , Moffitt , T. E. , & Hariri , A. R. ( 2019 ). White matter hyperintensities are common in midlife and already associated with cognitive decline . Brain Communications , 1 ( 1 ), fcz041 . 10.1093/braincomms/fcz041 31894208 PMC6928390

[b12] de Havenon , A. , Majersik , J. J. , Tirschwell , D. L. , McNally , J. S. , Stoddard , G. , & Rost , N. S. ( 2019 ). Blood pressure, glycemic control, and white matter hyperintensity progression in type 2 diabetics . Neurology , 92 ( 11 ), e1168 – e1175 . 10.1212/wnl.0000000000007093 30737332 PMC6511110

[b13] De Leeuw , F. E. , de Groot , J. C. , Achten , E. , Oudkerk , M. , Ramos , L. M. P. , Heijboer , R. , Hofman , A. , Jolles , J. , Van Gijn , J. , & Breteler , M. M. B. ( 2001 ). Prevalence of cerebral white matter lesions in elderly people: A population based magnetic resonance imaging study. The Rotterdam Scan Study . Journal of Neurology, Neurosurgery & Psychiatry , 70 ( 1 ), 9 – 14 . 10.1136/jnnp.70.1.9 11118240 PMC1763449

[b14] Dearborn , J. L. , Schneider , A. L. C. , Sharrett , A. R. , Mosley , T. H. , Bezerra , D. C. , Knopman , D. S. , Selvin , E. , Jack , C. R. , Coker , L. H. , & Alonso , A. ( 2015 ). Obesity, insulin resistance, and incident small vessel disease on magnetic resonance imaging: Atherosclerosis risk in communities study . Stroke , 46 ( 11 ), 3131 – 3136 . 10.1161/strokeaha.115.010060 26451022 PMC4624467

[b15] Debette , S. , Schilling , S. , Duperron , M.-G. , Larsson , S. C. , & Markus , H. S. ( 2019 ). Clinical significance of magnetic resonance imaging markers of vascular brain injury: A systematic review and meta-analysis . JAMA Neurology , 76 ( 1 ), 81 – 94 . 10.1001/jamaneurol.2018.3122 30422209 PMC6439887

[b16] Debette , S. , Seshadri , S. , Beiser , A. , Au , R. , Himali , J. J. , Palumbo , C. , Wolf , P. A. , & DeCarli , C. ( 2011 ). Midlife vascular risk factor exposure accelerates structural brain aging and cognitive decline . Neurology , 77 ( 5 ), 461 – 468 . 10.1212/WNL.0b013e318227b227 21810696 PMC3146307

[b17] Dickie , D. A. , Karama , S. , Ritchie , S. J. , Cox , S. R. , Sakka , E. , Royle , N. A. , Aribisala , B. S. , Hernández , M. V. , Maniega , S. M. , & Pattie , A. ( 2016 ). Progression of white matter disease and cortical thinning are not related in older community-dwelling subjects . Stroke , 47 ( 2 ), 410 – 416 . 10.1161/strokeaha.115.011229 26696646 PMC5633325

[b18] Duering , M. , Biessels , G. J. , Brodtmann , A. , Chen , C. , Cordonnier , C. , Leeuw , F.-E. de , Debette , S. , Frayne , R. , Jouvent , E. , Rost , N. S. , Telgte , A. ter , Salman , R. A.-S. , Backes , W. H. , Bae , H.-J. , Brown , R. , Chabriat , H. , Luca , A. D. , deCarli , C. , Dewenter , A. , … Wardlaw , J. M. ( 2023 ). Neuroimaging standards for research into small vessel disease—Advances since 2013 . The Lancet Neurology , 22 ( 7 ), 602 – 618 . 10.1016/S1474-4422(23)00131-X 37236211

[b19] Dufouil , C. , de Kersaint–Gilly , A. , Besancon , V. , Levy , C. , Auffray , E. , Brunnereau , L. , Alperovitch , A. , & Tzourio , C. ( 2001 ). Longitudinal study of blood pressure and white matter hyperintensities: The EVA MRI cohort . Neurology , 56 ( 7 ), 921 – 926 . 10.1212/wnl.56.7.921 11294930

[b20] Dufouil , C. , Seshadri , S. , & Chene , G. ( 2014 ). Cardiovascular risk profile in women and dementia . Journal of Alzheimer’s Disease , 42 Suppl 4 (1875-8908 (Electronic)), S353 – S363 . 10.3233/JAD-141629 25351109

[b21] Engel , C. , Wirkner , K. , Zeynalova , S. , Baber , R. , Binder , H. , Ceglarek , U. , Enzenbach , C. , Fuchs , M. , Hagendorff , A. , Henger , S. , Hinz , A. , Rauscher , F. G. , Reusche , M. , Riedel-Heller , S. G. , Röhr , S. , Sacher , J. , Sander , C. , Schroeter , M. L. , Tarnok , A. , … LIFE-Adult-Study working group . ( 2023 ). Cohort profile: The LIFE-adult-study . International Journal of Epidemiology , 52 ( 1 ), e66 – e79 . 10.1093/ije/dyac114 35640047 PMC9908058

[b22] Fatemi , F. , Kantarci , K. , Graff-Radford , J. , Preboske , G. M. , Weigand , S. D. , Przybelski , S. A. , Knopman , D. S. , Machulda , M. M. , Roberts , R. O. , Mielke , M. M. , Petersen , R. C. , Jack , C. R. , & Vemuri , P. ( 2018 ). Sex differences in cerebrovascular pathologies on FLAIR in cognitively unimpaired elderly . Neurology , 90 ( 6 ), e466 . 10.1212/WNL.0000000000004913 29343465 PMC5818016

[b23] Franklin , S. S. , Gustin , W. , Wong , N. D. , Larson , M. G. , Weber , M. A. , Kannel , W. B. , & Levy , D. ( 1997 ). Hemodynamic patterns of age-related changes in blood pressure . Circulation , 96 ( 1 ), 308 – 315 . 10.1161/01.CIR.96.1.308 9236450

[b24] Godin , O. , Tzourio , C. , Maillard , P. , Mazoyer , B. , & Dufouil , C. ( 2011 ). Antihypertensive treatment and change in blood pressure are associated with the progression of white matter lesion volumes: The Three-City (3C)–Dijon Magnetic Resonance Imaging Study . Circulation , 123 ( 3 ), 266 – 273 . 10.1161/circulationaha.110.961052 21220733

[b25] Gottesman Rebecca , F. , Coresh , J. , Catellier Diane , J. , Sharrett , A. R. , Rose Kathryn , M. , Coker Laura , H. , Shibata Dean , K. , Knopman David , S. , Jack Clifford , R. , & Mosley Thomas , H. ( 2010 ). Blood pressure and white-matter disease progression in a biethnic cohort . Stroke , 41 ( 1 ), 3 – 8 . 10.1161/STROKEAHA.109.566992 19926835 PMC2803313

[b26] Griffanti , L. , Jenkinson , M. , Suri , S. , Zsoldos , E. , Mahmood , A. , Filippini , N. , Sexton , C. E. , Topiwala , A. , Allan , C. , Kivimäki , M. , Singh-Manoux , A. , Ebmeier , K. P. , Mackay , C. E. , & Zamboni , G. ( 2018 ). Classification and characterization of periventricular and deep white matter hyperintensities on MRI: A study in older adults . NeuroImage , 170 , 174 – 181 . 10.1016/j.neuroimage.2017.03.024 28315460

[b27] Gustafson , D. , Lissner , L. , Bengtsson , C. , Bjorkelund , C. , & Skoog , I. ( 2004 ). A 24-year follow-up of body mass index and cerebral atrophy . Neurology , 63 ( 10 ), 1876 – 1881 . 10.1212/01.wnl.0000141850.47773.5f 15557505

[b28] Hajek , A. , & König , H.-H. ( 2020 ). Fear of dementia in the general population: Findings from the German Socio-Economic Panel (GSOEP) . Journal of Alzheimer’s Disease , 75 , 1135 – 1140 . 10.3233/JAD-200106 32390634

[b29] Hamilton , O. , Backhouse , E. V. , Janssen , E. , Jochems , A. C. C. , Maher , C. , Ritakari , T. E. , Stevenson , A. J. , Xia , L. , Deary , I. J. , & Wardlaw , J. M. ( 2021 ). Cognitive impairment in sporadic cerebral small vessel disease: A systematic review and meta-analysis . Alzheimer’s & Dementia , 17 ( 4 ), 665 – 685 . 10.1002/alz.12221 PMC8593445 33185327

[b30] Hamilton , O. , Cox , S. R. , Okely , J. A. , Conte , F. , Ballerini , L. , Bastin , M. E. , Corley , J. , Taylor , A. M. , Page , D. , Gow , A. J. , Muñoz Maniega , S. , Redmond , P. , Valdés-Hernández , M. del C. , Wardlaw , J. M. , & Deary , I. J. ( 2021 ). Cerebral small vessel disease burden and longitudinal cognitive decline from age 73 to 82: The Lothian birth cohort 1936 . Translational Psychiatry , 11 ( 1 ), 1 – 12 . 10.1038/s41398-021-01495-4 34226517 PMC8257729

[b31] Heidari , S. , Babor , T. F. , De Castro , P. , Tort , S. , & Curno , M. ( 2016 ). Sex and gender equity in research: Rationale for the SAGER guidelines and recommended use . Research Integrity and Peer Review , 1 ( 1 ), 1 – 9 . 10.1186/s41073-016-0007-6 29451543 PMC5793986

[b32] Higuchi , S. , Kabeya , Y. , & Kato , K. ( 2017 ). Visceral-to-subcutaneous fat ratio is independently related to small and large cerebrovascular lesions even in healthy subjects . Atherosclerosis , 259 , 41 – 45 . 10.1016/j.atherosclerosis.2017.03.001 28285092

[b33] Jansen , M. G. , Griffanti , L. , Mackay , C. E. , Anatürk , M. , Melazzini , L. , Lange , A.-M. G. de , Filippini , N. , Zsoldos , E. , Wiegertjes , K. , Leeuw , F.-E. de , Singh-Manoux , A. , Kivimäki , M. , Ebmeier , K. P. , & Suri , S. ( 2022 ). Association of cerebral small vessel disease burden with brain structure and cognitive and vascular risk trajectories in mid-to-late life . Journal of Cerebral Blood Flow & Metabolism , 42 ( 4 ), 600 – 612 . 10.1177/0271678X211048411 34610763 PMC8943617

[b34] Jiménez-Balado , J. , Corlier , F. , Habeck , C. , Stern , Y. , & Eich , T. ( 2022 ). Effects of white matter hyperintensities distribution and clustering on late-life cognitive impairment . Scientific Reports , 12 ( 1 ), 1955 . 10.1038/s41598-022-06019-8 35121804 PMC8816933

[b35] Jiménez-Sánchez , L. , Hamilton , O. K. L. , Clancy , U. , Backhouse , E. V. , Stewart , C. R. , Stringer , M. S. , Doubal , F. N. , & Wardlaw , J. M. ( 2021 ). Sex differences in cerebral small vessel disease: A systematic review and meta-analysis . Frontiers in Neurology , 12 , 756887 . 10.3389/fneur.2021.756887 34777227 PMC8581736

[b36] Jorgensen , D. R. , Shaaban , C. E. , Wiley , C. A. , Gianaros , P. J. , Mettenburg , J. , & Rosano , C. ( 2018 ). A population neuroscience approach to the study of cerebral small vessel disease in midlife and late life: An invited review . American Journal of Physiology-Heart and Circulatory Physiology , 314 ( 6 ), H1117 – H1136 . 10.1152/ajpheart.00535.2017 29393657 PMC6032084

[b37] Kaess , B. M. , Rong , J. , Larson , M. G. , Hamburg , N. M. , Vita , J. A. , Levy , D. , Benjamin , E. J. , Vasan , R. S. , & Mitchell , G. F. ( 2012 ). Aortic stiffness, blood pressure progression, and incident hypertension . JAMA , 308 ( 9 ), 875 – 881 . 10.1001/2012.jama.10503 22948697 PMC3594687

[b38] Kawas , C. H. , Corrada , M. M. , & Whitmer , R. A. ( 2021 ). Diversity and disparities in dementia diagnosis and care: A challenge for all of us . JAMA Neurology , 78 ( 6 ), 650 – 652 . 10.1001/jamaneurol.2021.0285 33779687

[b39] Kharabian Masouleh , S. , Arelin , K. , Horstmann , A. , Lampe , L. , Kipping , J. A. , Luck , T. , Riedel-Heller , S. G. , Schroeter , M. L. , Stumvoll , M. , Villringer , A. , & Witte , A. V. ( 2016 ). Higher body mass index in older adults is associated with lower gray matter volume: Implications for memory performance . Neurobiology of Aging , 40 , 1 – 10 . 10.1016/j.neurobiolaging.2015.12.020 26973099

[b40] Kim , K. W. , Seo , H. , Kwak , M. S. , & Kim , D. ( 2017 ). Visceral obesity is associated with white matter hyperintensity and lacunar infarct . International Journal of Obesity , 41 ( 5 ), 683 – 688 . 10.1038/ijo.2017.13 28104915

[b41] Kloppenborg , R. P. , Nederkoorn , P. J. , Geerlings , M. I. , & van den Berg , E. ( 2014 ). Presence and progression of white matter hyperintensities and cognition . Neurology , 82 ( 23 ), 2127 . 10.1212/WNL.0000000000000505 24814849

[b42] Kynast , J. , Lampe , L. , Luck , T. , Frisch , S. , Arelin , K. , Hoffmann , K.-T. , Loeffler , M. , Riedel-Heller , S. G. , Villringer , A. , & Schroeter , M. L. ( 2018 ). White matter hyperintensities associated with small vessel disease impair social cognition beside attention and memory . Journal of Cerebral Blood Flow & Metabolism , 38 ( 6 ), 996 – 1009 . 10.1177/0271678x17719380 28685621 PMC5999004

[b43] Lai , Y. , Jiang , C. , Du , X. , Sang , C. , Guo , X. , Bai , R. , Tang , R. , Dong , J. , & Ma , C. ( 2020 ). Effect of intensive blood pressure control on the prevention of white matter hyperintensity: Systematic review and meta-analysis of randomized trials . Journal of Clinical Hypertension (Greenwich, Conn.) , 22 ( 11 ), 1968 – 1973 . 10.1111/jch.14030 33459521 PMC8029786

[b44] Lampe , L. , Kharabian-Masouleh , S. , Kynast , J. , Arelin , K. , Steele , C. J. , Löffler , M. , Witte , A. V. , Schroeter , M. L. , Villringer , A. , & Bazin , P.-L. ( 2019 ). Lesion location matters: The relationships between white matter hyperintensities on cognition in the healthy elderly . Journal of Cerebral Blood Flow & Metabolism , 39 ( 1 ), 36 – 43 . 10.1177/0271678X17740501 29106319 PMC6311671

[b45] Lampe , L. , Zhang , R. , Beyer , F. , Huhn , S. , Kharabian Masouleh , S. , Preusser , S. , Bazin , P.-L. , Schroeter , M. L. , Villringer , A. , & Witte , A. V. ( 2019 ). Visceral obesity relates to deep white matter hyperintensities via inflammation . Annals of Neurology , 85 ( 2 ), 194 – 203 . 10.1002/ana.25396 30556596 PMC6590485

[b46] Lampert , T. , Kroll , L. , Müters , S. , & Stolzenberg , H. ( 2013 ). Messung des sozioökonomischen Status in der Studie zur Gesundheit Erwachsener in Deutschland (DEGS1) . Bundesgesundheitsblatt-Gesundheitsforschung-Gesundheitsschutz , 56 ( 5-6 ), 631 – 636 . 10.1007/s00103-012-1663-4 23703479

[b47] Livingston , G. , Huntley , J. , Sommerlad , A. , Ames , D. , Ballard , C. , Banerjee , S. , Brayne , C. , Burns , A. , Cohen-Mansfield , J. , Cooper , C. , Costafreda , S. G. , Dias , A. , Fox , N. , Gitlin , L. N. , Howard , R. , Kales , H. C. , Kivimäki , M. , Larson , E. B. , Ogunniyi , A. , … Mukadam , N. ( 2020 ). Dementia prevention, intervention, and care: 2020 report of the Lancet Commission . The Lancet , 396 ( 10248 ), 413 – 446 . 10.1016/s0140-6736(20)30367-6 PMC7392084 32738937

[b48] Loeffler , M. , Engel , C. , Ahnert , P. , Alfermann , D. , Arelin , K. , Baber , R. , Beutner , F. , Binder , H. , Brahler , E. , Burkhardt , R. , Ceglarek , U. , Enzenbach , C. , Fuchs , M. , Glaesmer , H. , Girlich , F. , Hagendorff , A. , Hantzsch , M. , Hegerl , U. , Henger , S. , … Thiery , J. ( 2015 ). The LIFE-Adult-study: Objectives and design of a population-based cohort study with 10,000 deeply phenotyped adults in Germany . BMC Public Health , 15 ( 1 ), 691 . 10.1186/s12889-015-1983-z 26197779 PMC4509697

[b49] Lohner , V. , Pehlivan , G. , Sanroma , G. , Miloschewski , A. , Schirmer , M. D. , Stöcker , T. , Reuter , M. , & Breteler , M. M. B. ( 2022 ). Relation between sex, menopause, and white matter hyperintensities: The Rhineland study . Neurology , 99 ( 9 ), e935 – e943 . 10.1212/WNL.0000000000200782 35768207 PMC9502737

[b90] Maillard , P. , Crivello , F. , Dufouil , C. , Tzourio-Mazoyer , N. , Tzourio , C. , & Mazoyer , B . ( 2009 ). Longitudinal follow-up of individual white matter hyperintensities in a large cohort of elderly . Neuroradiology , 51 ( 4 ), 209 – 220 . 19139875 10.1007/s00234-008-0489-0

[b50] Marini , S. , Anderson Christopher , D. , & Rosand , J. ( 2020 ). Genetics of cerebral small vessel disease . Stroke , 51 ( 1 ), 12 – 20 . 10.1161/STROKEAHA.119.024151 31752611 PMC7337039

[b95] Morey , R . ( 2017 ). One-tailed testing with lmBF . One-Tailed Testing with LmBF . https://gist.github.com/richarddmorey/7c1bd06a14384412f2145daee315c036

[b51] Morris , J. C. , Heyman , A. , Mohs , R. C. , Hughes , J. P. , Van Belle , G. , Fillenbaum , G. , Mellits , E. D. , & Clark , C. ( 1989 ). The consortium to establish a registry for Alzheimer’s disease (CERAD): I. Clinical and neuropsychological assessment of Alzheimer’s disease . Neurology , 39 ( 9 ), 1159 – 1165 . 10.1212/wnl.39.9.1159 2771064

[b52] Morys , F. , Dadar , M. , & Dagher , A. ( 2021 ). Association between mid-life obesity, its metabolic consequences, cerebrovascular disease and cognitive decline . The Journal of Clinical Endocrinology & Metabolism , 106 ( 10 ), e4260 – e4274 . 10.1210/clinem/dgab135 33677592 PMC8475210

[b53] Nasrallah , I. M. , Pajewski , N. M. , Auchus , A. P. , Chelune , G. , Cheung , A. K. , Cleveland , M. L. , Coker , L. H. , Crowe , M. G. , Cushman , W. C. , Cutler , J. A. , Davatzikos , C. , Desiderio , L. , Doshi , J. , Erus , G. , Fine , L. J. , Gaussoin , S. A. , Harris , D. , Johnson , K. C. , Kimmel , P. L. , … Bryan , R. N. ( 2019 ). Association of intensive vs standard blood pressure control with cerebral white matter lesions . JAMA , 322 ( 6 ), 524 – 534 . 10.1001/jama.2019.10551 31408137 PMC6692679

[b54] Nowok , B. , Raab , G. M. , & Dibben , C. ( 2016 ). Synthpop: Bespoke creation of synthetic data in R . Journal of Statistical Software , 74 ( 11 ), 1 – 26 . 10.18637/jss.v074.i11

[b55] Oosterman , J. M. , Vogels , R. L. C. , van Harten , B. , Gouw , A. A. , Poggesi , A. , Scheltens , P. , Kessels , R. P. C. , & Scherder , E. J. A. ( 2010 ). Assessing mental flexibility: Neuroanatomical and neuropsychological correlates of the trail making test in elderly people . The Clinical Neuropsychologist , 24 ( 2 ), 203 – 219 . 10.1080/13854040903482848 20162494

[b56] Peng , J. , Lu , F. , Wang , Z. , Zhong , M. , Sun , L. , Hu , N. , Liu , Z. , & Zhang , W. ( 2014 ). Excessive lowering of blood pressure is not beneficial for progression of brain white matter hyperintensive and cognitive impairment in elderly hypertensive patients: 4-year follow-up study . Journal of the American Medical Directors Association , 15 ( 12 ), 904 – 910 . 10.1016/j.jamda.2014.07.005 25239015

[b57] Pinto , E. ( 2007 ). Blood pressure and ageing . Postgraduate Medical Journal , 83 ( 976 ), 109 – 114 . 10.1136/pgmj.2006.048371 17308214 PMC2805932

[b58] Promjunyakul , N. , Dodge , H. H. , Lahna , D. , Boespflug , E. L. , Kaye , J. A. , Rooney , W. D. , & Silbert , L. C. ( 2018 ). Baseline NAWM structural integrity and CBF predict periventricular WMH expansion over time . Neurology , 90 ( 24 ), e2119 . 10.1212/WNL.0000000000005684 29769375 PMC5996835

[b59] Reuter , M. , Schmansky , N. J. , Rosas , H. D. , & Fischl , B. ( 2012 ). Within-subject template estimation for unbiased longitudinal image analysis . NeuroImage , 61 ( 4 ), 1402 – 1418 . 10.1016/j.neuroimage.2012.02.084 22430496 PMC3389460

[b60] Sachdev , P. , Parslow , R. , Wen , W. , Anstey , K. J. , & Easteal , S. ( 2009 ). Sex differences in the causes and consequences of white matter hyperintensities . Neurobiology of Aging , 30 ( 6 ), 946 – 956 . 10.1016/j.neurobiolaging.2007.08.023 17950492

[b61] Sachdev , P. , Wen , W. , Chen , X. , & Brodaty , H. ( 2007 ). Progression of white matter hyperintensities in elderly individuals over 3 years . Neurology , 68 ( 3 ), 214 – 222 . 10.1212/01.wnl.0000251302.55202.73 17224576

[b62] Scharf , E. L. , Graff-Radford , J. , Przybelski , S. A. , Lesnick , T. G. , Mielke , M. M. , Knopman , D. S. , Preboske , G. M. , Schwarz , C. G. , Senjem , M. L. , Gunter , J. L. , Machulda , M. , Kantarci , K. , Petersen , R. C. , Jack , C. R. , & Vemuri , P. ( 2019 ). Cardiometabolic health and longitudinal progression of white matter hyperintensity: The Mayo Clinic study of aging . Stroke , 50 ( 11 ), 3037 – 3044 . 10.1161/STROKEAHA.119.025822 31510903 PMC6817406

[b63] Schindler , L. S. , Subramaniapillai , S. , Ambikairajah , A. , Barth , C. , Crestol , A. , Voldsbekk , I. , Beck , D. , Gurholt , T. P. , Topiwala , A. , Suri , S. , Ebmeier , K. P. , Andreassen , O. A. , Draganski , B. , Westlye , L. T. , & de Lange , A.-M. G. ( 2023 ). Cardiometabolic health across menopausal years is linked to white matter hyperintensities up to a decade later . Frontiers in Global Women’s Health , 4 , 1320640 . 10.31219/osf.io/ksphx PMC10783171 38213741

[b64] Schmidt , P. , Pongratz , V. , Küster , P. , Meier , D. , Wuerfel , J. , Lukas , C. , Bellenberg , B. , Zipp , F. , Groppa , S. , Sämann , P. G. , Weber , F. , Gaser , C. , Franke , T. , Bussas , M. , Kirschke , J. , Zimmer , C. , Hemmer , B. , & Mühlau , M. ( 2019 ). Automated segmentation of changes in FLAIR-hyperintense white matter lesions in multiple sclerosis on serial magnetic resonance imaging . NeuroImage: Clinical , 23 , 101849 . 10.1016/j.nicl.2019.101849 31085465 PMC6517532

[b65] Schmidt , P. , & Wink , L. ( 2017 ). LST: A lesion segmentation tool for SPM . Manual/Documentation for Version , 2 , 15 . 10.2172/1046011

[b66] Schmidt , R. , Ropele , S. , Enzinger , C. , Petrovic , K. , Smith , S. , Schmidt , H. , Matthews , P. M. , & Fazekas , F. ( 2005 ). White matter lesion progression, brain atrophy, and cognitive decline: The Austrian stroke prevention study . Annals of Neurology , 58 ( 4 ), 610 – 616 . 10.1002/ana.20630 16178017

[b92] Shimokata , H. , Andres , R. , Coon , P. J. , Elahi , D. , Muller , D. C. , & Tobin , J. D . ( 1989 ). Studies in the distribution of body fat. II. Longitudinal effects of change in weight . International Journal of Obesity , 13 ( 4 ), 455 – 464 . 2676875

[b67] Somes , G. W. , Pahor , M. , Shorr , R. I. , Cushman , W. C. , & Applegate , W. B. ( 1999 ). The role of diastolic blood pressure when treating isolated systolic hypertension . Archives of Internal Medicine , 159 ( 17 ), 2004 – 2009 . 10.1001/archinte.159.17.2004 10510985

[b68] SPRINT Research Group , Wright , J. T. , Williamson , J. D. , Whelton , P. K. , Snyder , J. K. , Sink , K. M. , Rocco , M. V. , Reboussin , D. M. , Rahman , M. , Oparil , S. , Lewis , C. E. , Kimmel , P. L. , Johnson , K. C. , Goff , D. C. , Fine , L. J. , Cutler , J. A. , Cushman , W. C. , Cheung , A. K. , & Ambrosius , W. T. ( 2015 ). A randomized trial of intensive versus standard blood-pressure control . The New England Journal of Medicine , 373 ( 22 ), 2103 – 2116 . 10.1056/NEJMoa1511939 26551272 PMC4689591

[b69] Sudre , C. H. , Anson , B. G. , Davagnanam , I. , Schmitt , A. , Mendelson , A. F. , Prados , F. , Smith , L. , Atkinson , D. , Hughes , A. D. , & Chaturvedi , N. ( 2018 ). Bullseye’s representation of cerebral white matter hyperintensities . Journal of Neuroradiology , 45 ( 2 ), 114 – 122 . 10.1016/j.neurad.2017.10.001 29132940 PMC5867449

[b70] Thanprasertsuk , S. , Martinez-Ramirez , S. , Pontes-Neto , O. M. , Ni , J. , Ayres , A. , Reed , A. , Swords , K. , Gurol , M. E. , Greenberg , S. M. , & Viswanathan , A. ( 2014 ). Posterior white matter disease distribution as a predictor of amyloid angiopathy . Neurology , 83 ( 9 ), 794 – 800 . 10.1212/WNL.0000000000000732 25063759 PMC4155043

[b71] Veldsman , M. , Kindalova , P. , Husain , M. , Kosmidis , I. , & Nichols , T. E. ( 2020 ). Spatial distribution and cognitive impact of cerebrovascular risk-related white matter hyperintensities . NeuroImage: Clinical , 28 , 102405 . 10.1016/j.nicl.2020.102405 32971464 PMC7511743

[b93] Verhaaren , B. F. J. , Vernooij , M. W. , de Boer , R. , Hofman , A. , Niessen , W. J. , van der Lugt , A. , & Ikram , M. A . ( 2013 ). High blood pressure and cerebral white matter lesion progression in the general population . Hypertension , 61 ( 6 ), 1354 – 1359 . 10.1161/HYPERTENSIONAHA.111.00430 23529163

[b72] Verhaaren , B. F. J. , Debette , S. , Bis , J. C. , Smith , J. A. , Ikram , M. K. , Adams , H. H. , Beecham , A. H. , Rajan , K. B. , Lopez , L. M. , & Barral , S. ( 2015 ). Multiethnic genome-wide association study of cerebral white matter hyperintensities on MRI . Circulation: Cardiovascular Genetics , 8 ( 2 ), 398 – 409 . 10.1161/str.50.suppl_1.wp216 25663218 PMC4427240

[b73] Vermeer , S. E. , Longstreth , W. T. , & Koudstaal , P. J. ( 2007 ). Silent brain infarcts: A systematic review . The Lancet Neurology , 6 ( 7 ), 611 – 619 . 10.1016/S1474-4422(07)70170-9 17582361

[b74] Vuorinen , M. , Solomon , A. , Rovio , S. , Nieminen , L. , Kåreholt , I. , Tuomilehto , J. , Soininen , H. , & Kivipelto , M. ( 2011 ). Changes in vascular risk factors from midlife to late life and white matter lesions: A 20-year follow-up study . Dementia and Geriatric Cognitive Disorders , 31 ( 2 ), 119 – 125 . 10.1159/000323810 21273771

[b75] Walker , J. A. ( 2018 ). Applied statistics for experimental biology . Online Self-publication. https://www.middleprofessor.com/files/applied-biostatistics_bookdown/_book/

[b76] Wang , J.-G. , Staessen , J. A. , Franklin , S. S. , Fagard , R. , & Gueyffier , F. ( 2005 ). Systolic and diastolic blood pressure lowering as determinants of cardiovascular outcome . Hypertension , 45 ( 5 ), 907 – 913 . 10.1161/01.HYP.0000165020.14745.79 15837826

[b77] Wardlaw , J. M. , Debette , S. , Jokinen , H. , De Leeuw , F.-E. , Pantoni , L. , Chabriat , H. , Staals , J. , Doubal , F. , Rudilosso , S. , Eppinger , S. , Schilling , S. , Ornello , R. , Enzinger , C. , Cordonnier , C. , Taylor-Rowan , M. , & Lindgren , A. G. ( 2021 ). ESO Guideline on covert cerebral small vessel disease . European Stroke Journal , 6 ( 2 ), CXI – CLXII . 10.1177/23969873211012132 34414301 PMC8370079

[b78] Wardlaw , J. M. , Smith , C. , & Dichgans , M. ( 2019 ). Small vessel disease: Mechanisms and clinical implications . The Lancet Neurology , 18 ( 7 ), 684 – 696 . 10.1016/S1474-4422(19)30079-1 31097385

[b79] Wartolowska , K. A. , & Webb , A. J. S. ( 2021 ). Midlife blood pressure is associated with the severity of white matter hyperintensities: Analysis of the UK Biobank cohort study . European Heart Journal , 42 ( 7 ), 750 – 757 . 10.1093/eurheartj/ehaa756 33238300 PMC7882359

[b80] Wen , W. , Sachdev , P. S. , Li , J. J. , Chen , X. , & Anstey , K. J. ( 2009 ). White matter hyperintensities in the forties: Their prevalence and topography in an epidemiological sample aged 44–48 . Human Brain Mapping , 30 ( 4 ), 1155 – 1167 . 10.1002/hbm.20586 18465744 PMC6870596

[b81] Wilkinson , I. , & Webb , A. J. S. ( 2022 ). Consistency of associations of systolic and diastolic blood pressure with white matter hyperintensities: A meta-analysis . International Journal of Stroke , 17 ( 3 ), 291 – 298 . 10.1177/17474930211043364 34427478 PMC8864334

[b82] Williamson , W. , Lewandowski , A. J. , Forkert , N. D. , Griffanti , L. , Okell , T. W. , Betts , J. , Boardman , H. , Siepmann , T. , McKean , D. , & Huckstep , O. ( 2018 ). Association of cardiovascular risk factors with MRI indices of cerebrovascular structure and function and white matter hyperintensities in young adults . JAMA , 320 ( 7 ), 665 – 673 . 10.1001/jama.2018.11498 30140877 PMC6142949

[b91] Wills , A. K. , Lawlor , D. A. , Matthews , F. E. , Aihie Sayer , A. , Bakra , E. , Ben-Shlomo , Y. , Benzeval , M. , Brunner , E. , Cooper , R. , & Kivimaki , M . ( 2011 ). Life course trajectories of systolic blood pressure using longitudinal data from eight UK cohorts . PLoS Medicine , 8 ( 6 ), e1000440 . 21695075 10.1371/journal.pmed.1000440 PMC3114857

[b83] Yamashiro , K. , Tanaka , R. , Tanaka , Y. , Miyamoto , N. , Shimada , Y. , Ueno , Y. , Urabe , T. , & Hattori , N. ( 2014 ). Visceral fat accumulation is associated with cerebral small vessel disease . European Journal of Neurology , 21 ( 4 ), 667 – 673 . 10.1111/ene.12374 24495037

[b84] Zhang , D. , Tang , Y. , Ge , J. , Liu , Y. , Jin , J. , & He , M. ( 2020 ). Age and diastolic blood pressure play an important role in the progression of white matter lesions: A meta-analysis . European Neurology , 83 ( 4 ), 351 – 359 . 10.1159/000510077 32906133

[b85] Zhang , H. , Cui , Y. , Zhao , Y. , Dong , Y. , Duan , D. , Wang , J. , Sheng , L. , Ji , T. , Zhou , T. , & Hu , W. ( 2019 ). Effects of sartans and low-dose statins on cerebral white matter hyperintensities and cognitive function in older patients with hypertension: A randomized, double-blind and placebo-controlled clinical trial . Hypertension Research , 42 ( 5 ), 717 – 729 . 10.1038/s41440-018-0165-7 30552406

