# Supplementary material for: Progression of white matter hyperintensities is related to blood pressure increases and global cognitive decline – A registered report
Source: Imaging Neurosci (Camb). 2024 Jun 24;2:imag-2-00188. doi: 10.1162/imag_a_00188 (PMC12272209; doi:10.1162/imag_a_00188)
Supplement: Supplementary Material [file imag_a_00188-supp.pdf]

Supplements for Progression of white matter hyperintensities is related to blood pressure increases and global cognitive decline – a registered report

Frauke Beyer[1,2]

Laurenz Lammer[2]

Markus Loeffler[4,5]

Steffi Riedel-Heller[5,6]

Stéphanie Debette[1]

Arno Villringer[2,7]

A. Veronica Witte[2,3,7]

1 Bordeaux Population Health Research Center, University of Bordeaux, Inserm, UMR 1219, Bordeaux, France
2 Department of Neurology, Max Planck Institute for Human Cognitive and Brain Sciences, Leipzig
3 CRC 1052 “Obesity Mechanisms”, Subproject A1, University of Leipzig
4 Institute for Medical Informatics, Statistics and Epidemiology; University of Leipzig
5 Leipzig Research Centre for Civilisation Diseases (LIFE), Leipzig
6 Institute of Social Medicine, Occupational Health and Public Health, University of Leipzig , Leipzig
7 Day Clinic for Cognitive Neurology, University Hospital Leipzig, University of Leipzig

Missing data pattern

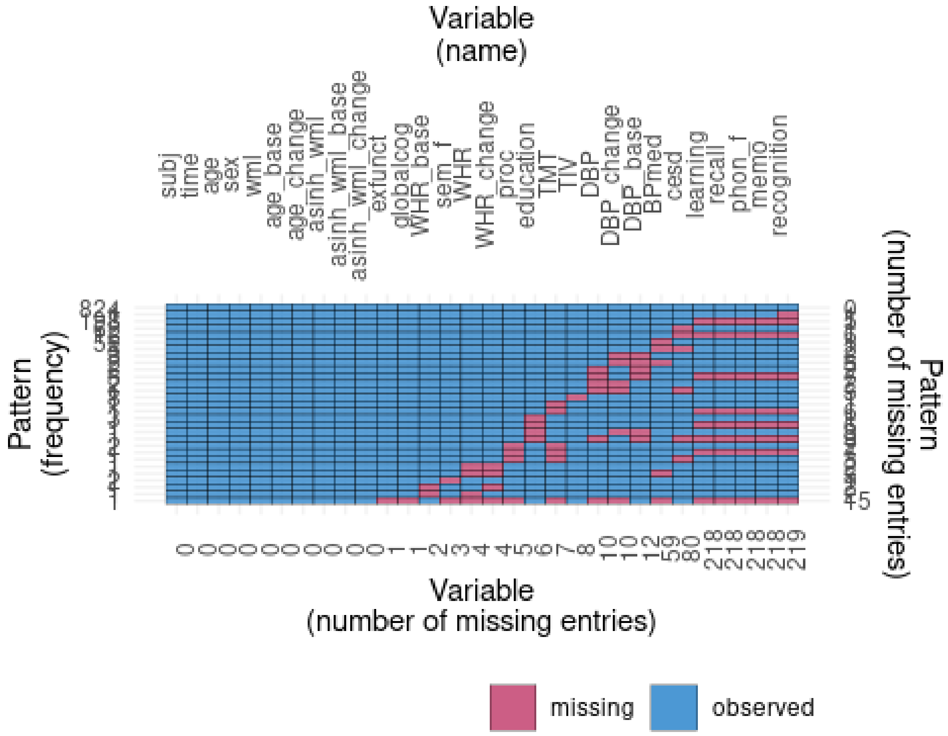

Comparison to a change score model

We compared our results from the mixed effect model with the approach taken in Debette et al. (2011) using change in raw WMH volume or asinh-transformed WMH volume as outcome measure in a linear model (see Table 1). In the change score model with raw WMH volume as outcome, higher DBP at baseline was associated with WMH progression, this was attenuated when using asinh-transformed WMH as outcome. Comparing the model assumptions of the original model (Figure 1 with the change score models for raw and asinh-transformed WMH (Figures 2 and 3), we noticed that the assumptions are not well fulfilled for the change score model.

Table 1: Results for DBP baseline and change from linear models using change in raw WMH or asinh-transformed WMH as outcome

	Raw WMH: Estimate [95 % CI]	Raw WMH: p-value	asinh WMH: Estimate [95 % CI]	asinh WMH: p-value
Age at baseline	0.102 [0.081, 0.122]	<0.001	0.003 [0.002, 0.003]	<0.001
Time between baseline and followup	-0.080 [-0.361, 0.201]	0.576	-0.002 [-0.009, 0.004]	0.491
DBP at baseline baseline	0.056 [0.035, 0.076]	<0.001	0.001 [0.000, 0.001]	0.030
Change in DBP	0.050 [0.030, 0.070]	<0.001	0.001 [0.001, 0.002]	<0.001

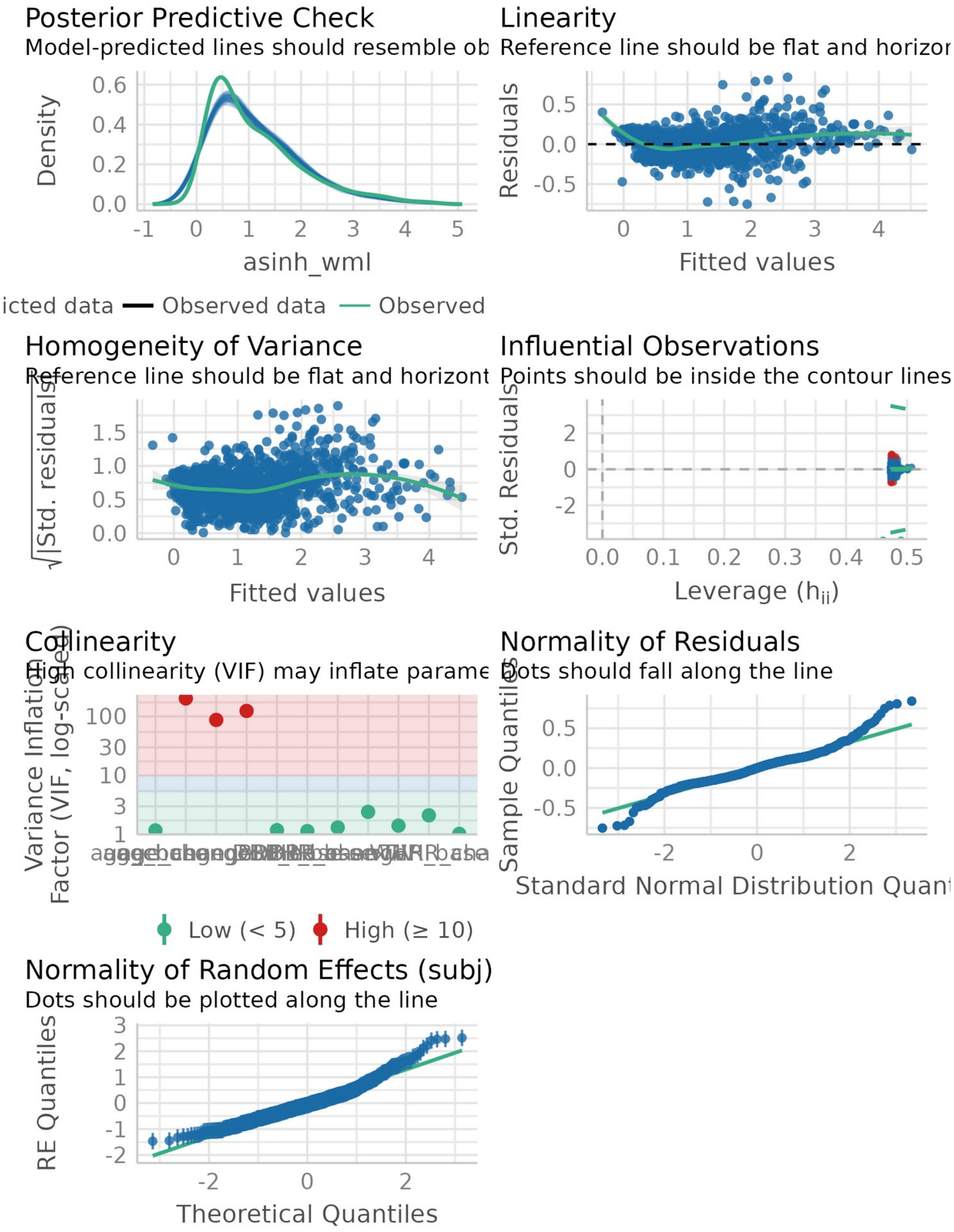

Figure 1: Assumptions of linear model for the original model M1 (from the package check_model)

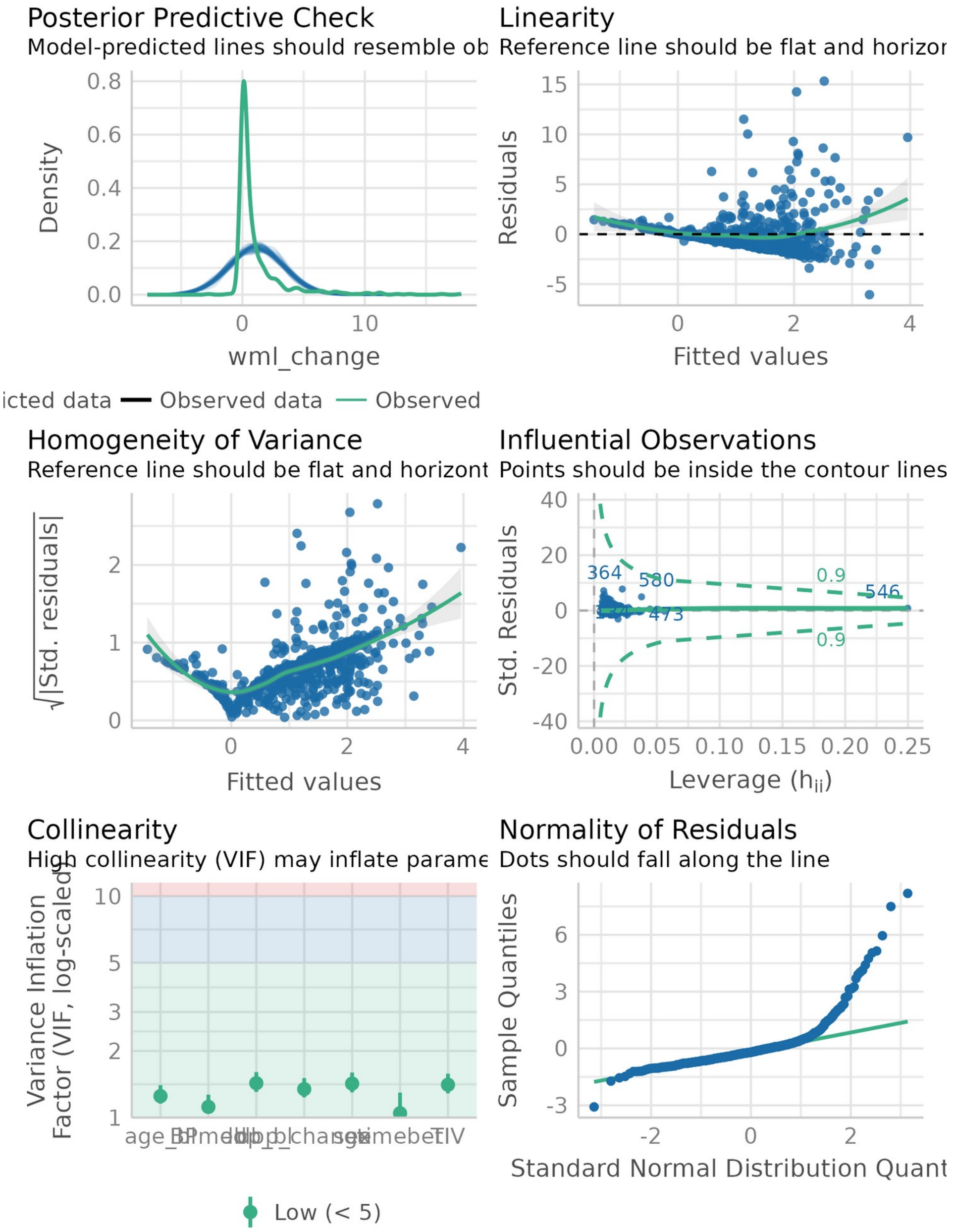

Figure 2: Assumptions of linear model for the change score model using raw WMH volume as outcome (from the package check_model)

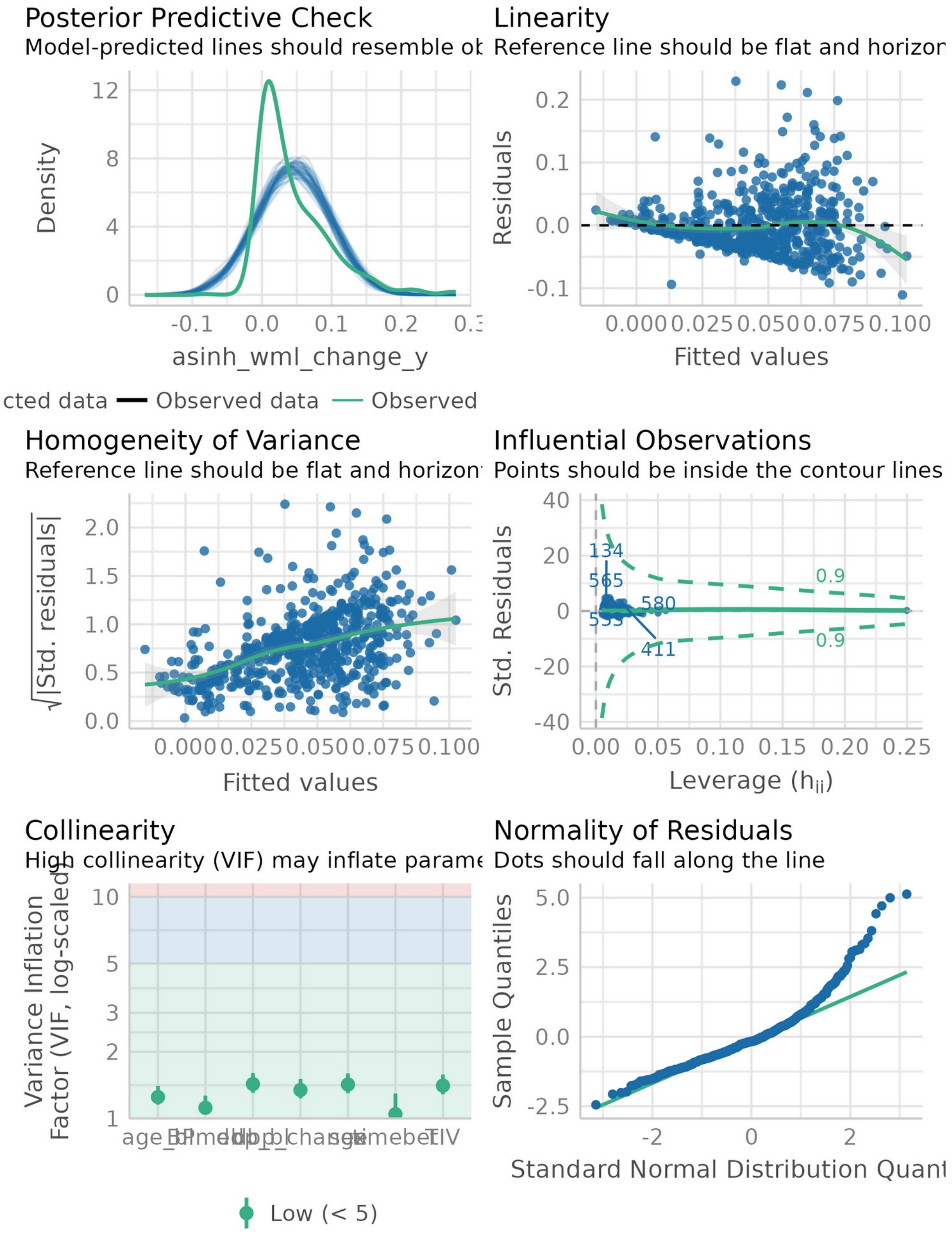

Figure 3: Assumptions of linear model for the change score model using asinh-transformed WMH volume as outcome (from the package check_model)

Assumptions for models M2 and M3

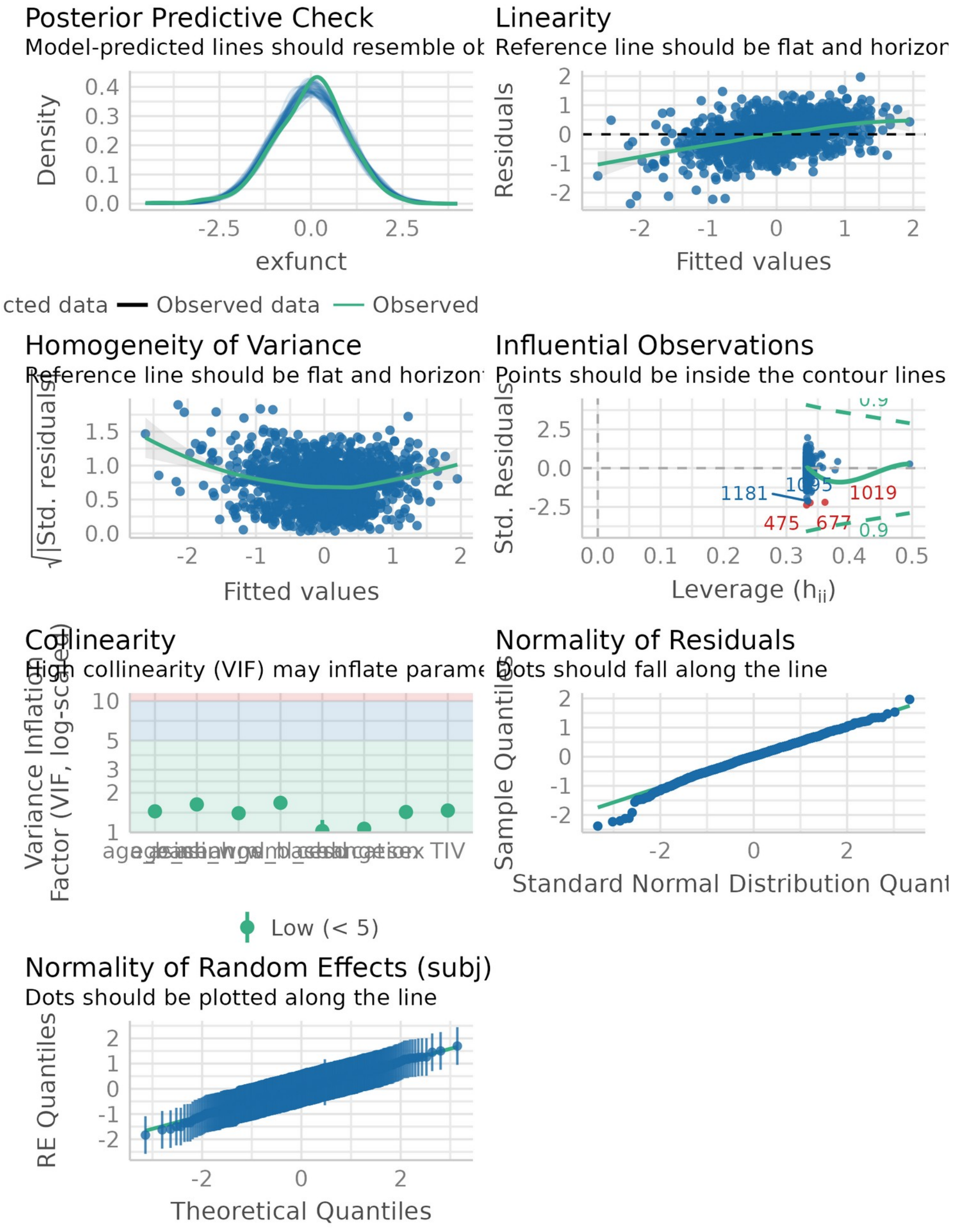

Figure 4: Assumptions of linear model for them model M2 (executive function)(from the package check_model)

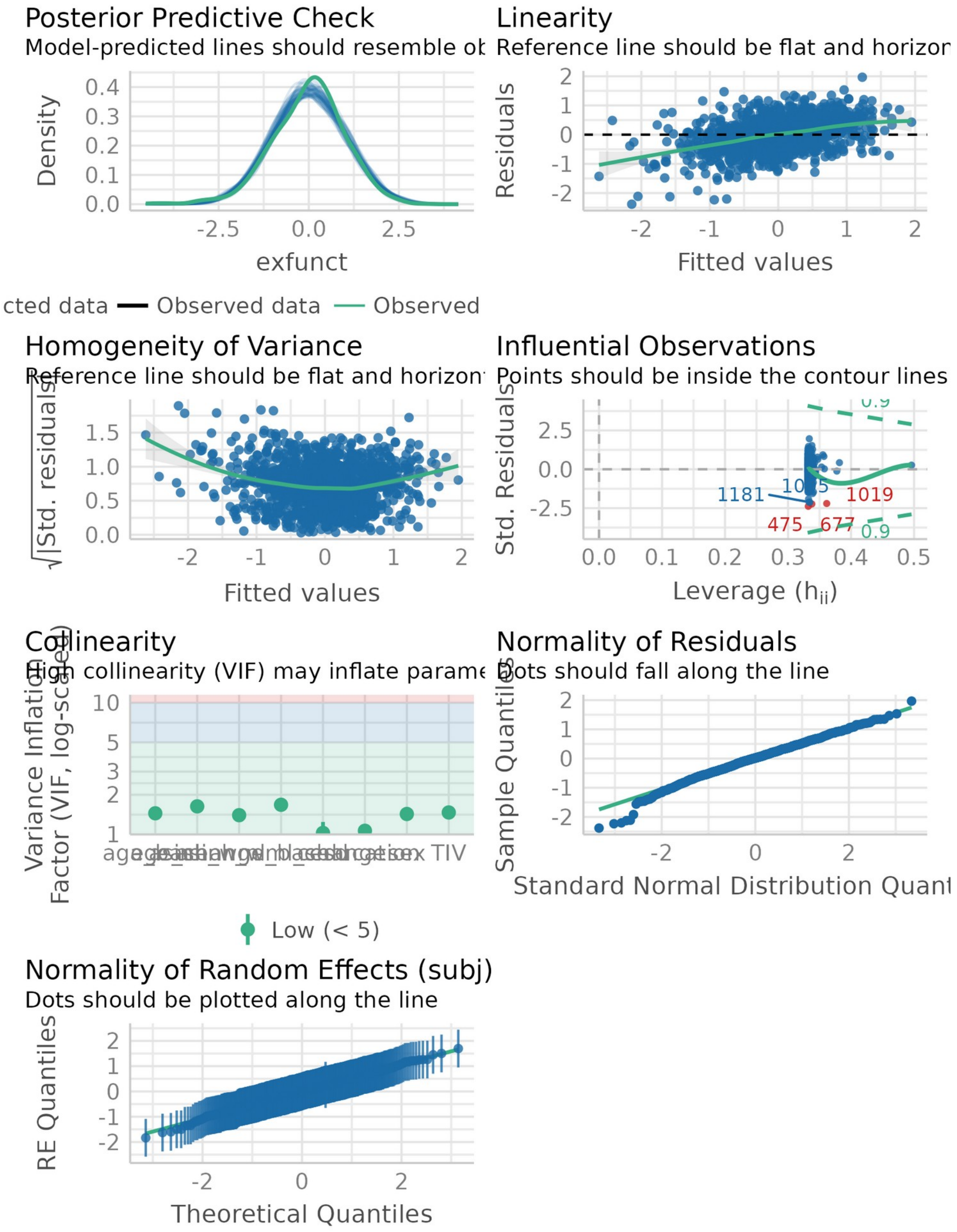

Figure 5: Assumptions of linear model for them model M3 (global cognitive function)(from the package check_model)

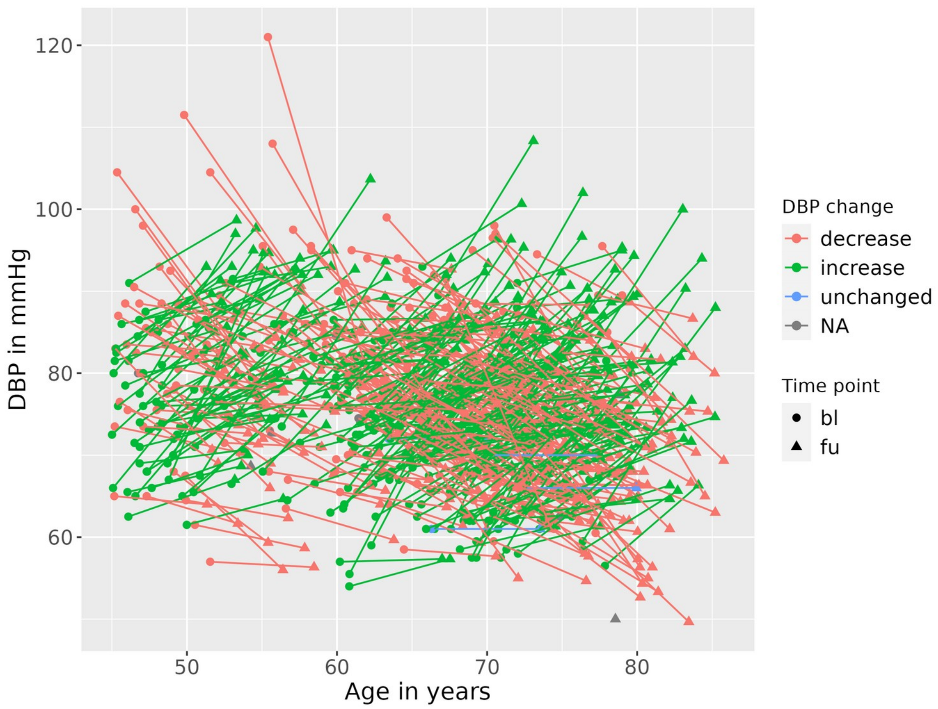

Figure 6: Age and DBP in Life-Adult. Colors indicate increase/stable/decrease in DBP over time.

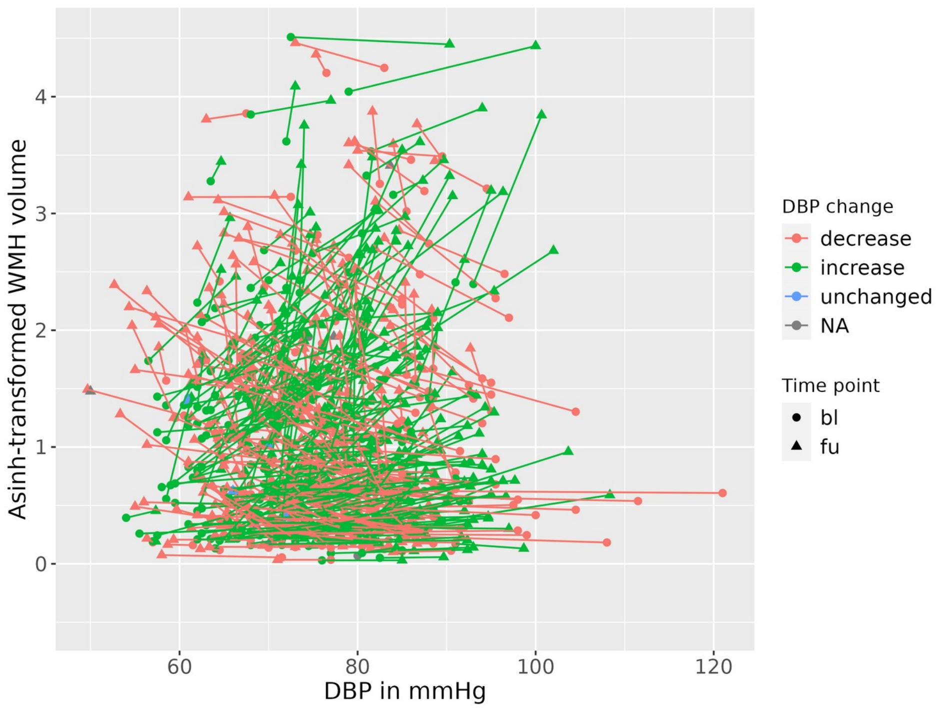

Figure 7: DBP and asinh-transformed WMH in Life-Adult. Colors indicate increase/stable/decrease in DBP over time.

Interaction Models

Table 2: Interaction of gender and WMH progression

	Estimate [95 % CI]	p-value
Age at baseline	0.056 [0.049, 0.062]	<0.001
Time between baseline and followup	0.025 [-0.045, 0.094]	0.486
Diastolic BP at baseline	0.012 [0.005, 0.018]	<0.001
Change in diastolic BP	0.006 [0.003, 0.009]	<0.001
Waist-to-hip ratio at baseline	0.836 [-0.134, 1.807]	0.091
Change in WHR	-0.078 [-0.693, 0.536]	0.803
Gender (males=1)	-0.281 [-0.462, -0.101]	0.002
Interaction of time and gender	0.000 [-0.012, 0.011]	0.936
Interaction of time and DBP at baseline	0.000 [-0.001, 0.000]	0.616
Interaction of time and WHR at baseline	0.031 [-0.035, 0.098]	0.356

E2aa: Gender and DBP change interaction (new exploratory analysis)

Table 3: Interaction of DBP change and gender on WMH progression

	Estimate [95 % CI]	p-value
Age at baseline	0.056 [0.049, 0.062]	<0.001
Time between baseline and followup	0.026 [-0.030, 0.083]	0.359
Diastolic BP at baseline	0.012 [0.005, 0.018]	<0.001
Change in diastolic BP	0.008 [0.003, 0.013]	0.002
Waist-to-hip ratio at baseline	0.843 [-0.116, 1.802]	0.085
Change in WHR	-0.074 [-0.674, 0.526]	0.809
Gender (males=1)	-0.282 [-0.459, -0.105]	0.002
Interaction of DBP change and gender	-0.003 [-0.009, 0.003]	0.372
Interaction of time and DBP at baseline	0.000 [-0.001, 0.000]	0.586

E2a: Gender and baseline DBP interaction on WML progression

Table 4: Interaction of DBP baseline, age and gender on WML progression

	Estimate [95 % CI]	p-value
Age at baseline	0.055 [0.049, 0.062]	<0.001
Time between baseline and followup	0.014 [-0.064, 0.091]	0.730
Diastolic BP at baseline	0.015 [0.006, 0.025]	0.002
Change in diastolic BP	0.006 [0.003, 0.009]	<0.001
Waist-to-hip ratio at baseline	0.837 [-0.128, 1.802]	0.089
Change in WHR	-0.092 [-0.704, 0.520]	0.767
Gender (males=1)	0.208 [-0.768, 1.184]	0.676
Interaction of DBP and gender	-0.006 [-0.019, 0.006]	0.318
Interaction of time and gender	0.021 [-0.046, 0.089]	0.534
Interaction of time and DBP at baseline	0.000 [-0.001, 0.001]	0.932
Interaction of time and WHR at baseline	0.031 [-0.035, 0.097]	0.361
Gender differences of time x DBP at baseline	0.000 [-0.001, 0.001]	0.519

E2c: Interaction of gender and baseline WHR on WMH progression

Table 5: Interaction of gender and baseline WHR on WMH progression

	Estimate [95 % CI]	p-value
Age at baseline	0.056 [0.049, 0.062]	<0.001
Time between baseline and followup	0.052 [-0.045, 0.148]	0.295
Diastolic BP at baseline	0.012 [0.005, 0.018]	<0.001
Change in diastolic BP	0.006 [0.003, 0.009]	<0.001
Waist-to-hip ratio at baseline	0.705 [-0.745, 2.155]	0.340
Change in WHR	-0.118 [-0.736, 0.500]	0.708
Gender (males=1)	-0.493 [-2.254, 1.268]	0.583
Interaction of WHR and gender	0.228 [-1.662, 2.119]	0.813
Interaction of time and gender	-0.049 [-0.171, 0.073]	0.433
Interaction of time and DBP at baseline	0.000 [-0.001, 0.000]	0.618
Interaction of time and WHR at baseline	0.000 [-0.102, 0.102]	0.994
Gender differences of time x WHR at baseline	0.052 [-0.079, 0.184]	0.436

E3a: Interaction of gender and WMH progression on executive cognitive function

Table 6: Interaction of WMH progression and gender on executive function

	Estimate [95 % CI]	p-value
Age at baseline	-0.017 [-0.026, -0.008]	<0.001
Time between baseline and followup	-0.047 [-0.061, -0.033]	<0.001
Asinh-transformed WMH volume baseline	-0.017 [-0.116, 0.082]	0.735
Change in Asinh-transformed WMH volume	-0.097 [-0.370, 0.175]	0.483
Gender (males=1)	-0.229 [-0.392, -0.066]	0.006
Interaction of gender and Asinh-transformed WMH change	-0.104 [-0.417, 0.209]	0.513

Table 7: Interaction of gender and WMH progression on general cognitive function

	Estimate [95 % CI]	p-value
Age at baseline	-0.045 [-0.053, -0.037]	<0.001
Time between baseline and followup	-0.040 [-0.052, -0.028]	<0.001
Asinh-transformed WMH volume baseline	-0.044 [-0.135, 0.046]	0.337
Change in Asinh-transformed WMH volume	-0.231 [-0.463, 0.002]	0.052
Gender (males=1)	-0.273 [-0.422, -0.125]	<0.001
Interaction of gender and Asinh-transformed WMH change	-0.160 [-0.427, 0.107]	0.239

Supplementary Table 8: Constructs & Variables from LIFE-Adult

Construct	Variable	LIFE-Adults Questionnaire name and field	Timepoint for which measure is available
		**Variables for exclusion**	
Conditions to ensure we are looking at a healthy sample	Life-time diagnosis of MS, PD, epilepsy	Medical anamnese (T00173) MEDANAM_F0171, MEDANAM_F0179, MEDANAM_F0167	Baseline
		Medical anamnese (T01228) MEDIZ_AN_F26 MEDIZ_AN_F27 MEDIZ_AN_F31 (free field, used to screen for epilepsy)	Followup
	Previous stroke	Medical anamnese (T00173)	Baseline
		Cardiovascular anamnese (T01226)	Followup
		Lesion diagnosed by radiologist (ischemic, hemorraghic, traumatic lesions)	Baseline & Followup
	Incidental findings/non-usability of MRI	Radiologist's rating ("non usable") Radiologist's rating (incidental finding)	Baseline
		Radiologist's rating ("non usable") Radiologist's rating (incidental finding)	Followup
	Dementia or cognitive impairment	T00043 SIDAM MMSE < 24	Baseline
		T00043 SIDAM MMSE < 24 or dementia diagnosis in medical anamnese	Followup

	Intake of centrally active medication	(T01228)<br>MEDIZ_AN_F30<br>D00038<br>ATC codes/groups:<br>**M03B** MUSCLE RELAXANTS, CENTRALLY ACTING AGENTS<br>**N02A** OPIOIDS<br>**N03** ANTIEPILEPTICS<br>**N04** ANTI-PARKINSON DRUGS<br>**N05** PSYCHOLEPTICS<br>**N06A** ANTIDEPRESSANTS<br>**N06B** PSYCHOSTIMULANTS, AGENTS USED FOR ADHD AND NOOTROPICS<br>**N06D** ANTI-DEMENTIA DRUGS (except for N06DX02, Ginkgo folium)<br>**N07A** PARASYMPATHOMIMETICS	Baseline<br>Followup
**Used for**		**Covariates**	
All analyses	Baseline Age	Age at first MRI or cognitive assessment	Baseline
	Age Change	Time between first and second MRI assessment	Baseline<br>Followup
	Gender	self-reported binary gender	Baseline
	eTIV	Estimated total intracranial volume	Summary value of Baseline and Followup
Model M1	Hypertensive medication	Medical (T00173) or medication anamnese (D00038)<br>Intake of anti-hypertensive medication (ATC code starting with "C02 "C03", "C07", "C08" "C09")<br>or treatment because of hypertension	Baseline

		(MEDANAM_F0039)	
		Medication or cardiovascular Anamnese (T01226) Intake of anti-hypertensive medication (ATC code starting with "C02 "C03", "C07", "C08" "C09") or treatment because of hypertension (KARD_AN_F10_3)	Followup
Model M2	CES-D (log-transformed)	D00041 CES_D_SCORE_SUM _CES_D	Baseline
		T00013 CES_D_SCORE_SUM _CES_D	Followup
	Education	socioeconomic status (D00140) Binary variable based on whether participant has tertiary degree (SES2_sesbldg)	Baseline
		**Predictors of interest**	
Blood pressure	Systolic/ diastolic blood pressure	T00049 Average of three consecutive measurements	Baseline
		T01170 Average of three consecutive measurements	Followup
Visceral adiposity	Waist-to-hip ratio	D00074 Anthropometry	Baseline
		T01169 Anthropometry	Followup
Cerebral small vessel disease	WML (asinh-transformed)	From FLAIR imaging	Baseline Followup
	Voxelwise probability of new WML		
Memory function	Cerad learning & delayed recall & recognition	T00044 CERAD-plus test battery	Baseline

		T00044 CERAD-plus test battery	Followup
Executive function	CERAD verbal fluency (S and animals)	T00044 CERAD-plus test battery	Baseline
		T00042 CERAD Animals	
	TMTB/TMTA	T00041 TMT	Followup
Processing speed	TMTA	T00041 TMT	Baseline
		T00041 TMT	Followup

References

Debette, S., S. Seshadri, A. Beiser, R. Au, J. J. Himali, C. Palumbo, P. A. Wolf, and C. DeCarli. 2011. "Midlife Vascular Risk Factor Exposure Accelerates Structural Brain Aging and Cognitive Decline." *Neurology* 77 (5): 461–68. https://doi.org/10.1212/WNL.0b013e318227b227.
